# Reshaping the epigenetic landscape during early flower development: induction of attractor transitions by relative differences in gene decay rates

**DOI:** 10.1186/s12918-015-0166-y

**Published:** 2015-05-13

**Authors:** Jose Davila-Velderrain, Carlos Villarreal, Elena R Alvarez-Buylla

**Affiliations:** Instituto de Ecología, Universidad Nacional Autónoma de México, Cd. Universitaria, México, 04510 D.F. México; Centro de Ciencias de la Complejidad (C3), Universidad Nacional Autónoma de México, Cd. Universitaria, México, 04510 D.F. México; Instituto de Física, Universidad Nacional Autónoma de México, Cd. Universitaria, México, 04510 D.F. México

**Keywords:** Gene regulatory network, Epigenetic landscape, Attractor landscape, Differentiation, Flower development, Attractor transitions

## Abstract

**Background:**

Gene regulatory network (GRN) dynamical models are standard systems biology tools for the mechanistic understanding of developmental processes and are enabling the formalization of the epigenetic landscape (EL) model.

**Methods:**

In this work we propose a modeling framework which integrates standard mathematical analyses to extend the simple GRN Boolean model in order to address questions regarding the impact of gene specific perturbations in cell-fate decisions during development.

**Results:**

We systematically tested the propensity of individual genes to produce qualitative changes to the EL induced by modification of gene characteristic decay rates reflecting the temporal dynamics of differentiation stimuli. By applying this approach to the flower specification GRN (FOS-GRN) we uncovered differences in the functional (dynamical) role of their genes. The observed dynamical behavior correlates with biological observables. We found a relationship between the propensity of undergoing attractor transitions between attraction basins in the EL and the direction of differentiation during early flower development - being less likely to induce up-stream attractor transitions as the course of development progresses. Our model also uncovered a potential mechanism at play during the transition from EL basins defining inflorescence meristem to those associated to flower organs meristem. Additionally, our analysis provided a mechanistic interpretation of the homeotic property of the ABC genes, being more likely to produce both an induced inter-attractor transition and to specify a novel attractor. Finally, we found that there is a close relationship between a gene’s topological features and its propensity to produce attractor transitions.

**Conclusions:**

The study of how the state-space associated with a dynamical model of a GRN can be restructured by modulation of genes’ characteristic expression times is an important aid for understanding underlying mechanisms occurring during development. Our contribution offers a simple framework to approach such problem, as exemplified here by the case of flower development. Different GRN models and the effect of diverse inductive signals can be explored within the same framework. We speculate that the dynamical role of specific genes within a GRN, as uncovered here, might give information about which genes are more likely to link a module to other regulatory circuits and signaling transduction pathways.

**Electronic supplementary material:**

The online version of this article (doi:10.1186/s12918-015-0166-y) contains supplementary material, which is available to authorized users.

## Background

The *systems* perspective to biology has successfully rephrased long-standing questions in developmental biology in terms of the dynamical behavior of molecular networks [1-4]. A salient example is the increasing use of gene regulatory network (GRN) models to study cell-fate specification [5-9]. How can cells with the same genotype and gene regulatory network in multicellular organisms attain different cell fates? How are the steady-state gene expression configurations that characterize each cell-type attained? Why do we observe certain cellular phenotypes and not others? How are the temporal and spatial patterns of cell-fate decisions established and how are they robustly maintained? The dynamical analysis of GRNs has given insights into these and other important questions concerning cell differentiation and morphogenesis, the two components of development. In short, GRN models are showing how observed differentiation patterns can be understood in mechanistic terms [10]. Overall, experimentally grounded GRN models constitute multistable dynamical systems able to recover stable steady states (or *attractors*) corresponding to fixed profiles of gene activation that mimic those characterizing different cell types in both plants and animals (e.g., [11,12]). Such profiles are commonly interpreted as cell fates [1,4,13].

The first, and arguably the simplest, model of GRN dynamics is the Boolean network model proposed by Stuart Kauffman [14]. This model is based on strong assumptions, mainly: (1) gene activity shows binary (on/off) behavior; (2) the temporal change in gene activity occurs in discrete, regular steps; and, originally, (3) the activity state of the whole network evolves in a synchronized manner [15]. Albeit highly abstract at first sight, the applicability of Boolean GRNs, as well as derived conceptual implications, have been supported extensively both by experimental observations [5,16,17] and by theoretical GRNs grounded on experimental data [11,18]. A first example of the latter was proposed to understand cell-fate attainment during early flower development [19]. The Boolean GRN model has become a well established modeling tool in systems biology that is intuitive and attractive to biologists [20,21].

In addition, simple GRN dynamical models are enabling the formalization of old biology metaphors such as the conceptual model of the epigenetic landscape (EL) proposed by C.H. Waddington in 1950s [22-25]. In modern post-genomic biology the EL has been consolidated as the preferred conceptual framework for the discussion of the mechanistic basis underlying cellular differentiation and plasticity [26-28]. A formal basis for this metaphorical EL is being developed in the context of GRNs [24,29-32]. The key for this formalization is to consider that, as well as generating the cellular phenotypic sates (attractors), the GRN dynamics also partitions the whole state-space – the abstract space containing all the possible states of a given system – in specific regions restricting the trajectories from one state to another one. The formalization of the EL in this context is conceptually straightforward: the number, depth, width, and relative position of the attractor’s basins of attraction would correspond to the hills and valleys of the metaphorical EL [24]. Here, we refer to the structured order of the basins in state-space as the attractors landscape (AL). For our purposes, the characterization of an AL would correspond, in practical terms, to the characterization of an EL (see below). There is an increasing interest to model the EL associated with a GRN [9,24,30,33-37].

Despite developments in both the conceptual and technical aspects of GRN modeling, interest in novel questions associated with developmental cell plasticity calls for extended modeling frameworks. For example, previous modeling approaches are not able to address the importance of quantitative alterations of the GRN components in attractors (cell-fates) attainment and transitions, or the importance of particular GRN components in moving the system from a particular steady-state or cell fate to another one. In an attempt to contribute to such a need, in this work we propose a modeling framework that integrates standard dynamical systems analyses to extend the simple GRN Boolean model in order to address questions regarding the impact of gene specific perturbations in cell-fate decisions during development. Two different, non-exclusive, approaches are commonly followed in the study of GRN developmental dynamics: (1) analyzing a large set of randomly (or exhaustively) assembled networks (see, for example [38-40]); or (2) focusing on one, well-characterized and experimentally grounded GRN [11,18]. In this work we adopt the second approach.

One of the first GRN models, which is experimentally grounded and has been extensively validated and used to test different approaches, is the floral organ specification GRN (FOS-GRN). The GRN model proposes a regulatory module underlying floral organ determination in *Arabidopsis thaliana* during early stages of flower development [11,19,41]. The network is grounded in experimental data for 15 genes and their interactions. Among the 15 genes, five are grouped into three classes (A-type, B-type, and C- type), whose combinations have been shown - through molecular developmental genetic studies - to be necessary for floral organ cell specification. A-type genes (AP1 and AP2) are required for sepal identity, A-type together with B-type (AP3 and PI) for petal identity, B-type and C-type (AGAMOUS) for stamen identity, and the C-type gene (AG) alone for carpel primordia cell identity. The so-called ABC model describes such combinatorial activities during floral organ determination [42]. The original Boolean FOS-GRN converges to ten attractors that correspond to the main cell types observed during early flower development, and thus provided a mechanistic explanation to the ABC model. Six attractors correspond to sepal (Sep), petal (Pt1 and Pt2), stamen (St1 and St2), and carpel (Car) primordial cells within flower meristems with the expected ABC gene combinations for each floral organ primordi. In addition it explained the configurations that characterize the inflorescence meristem: four attractors correspond to meristematic cells of the inflorescence, which is partitioned into four regions (Inf1, Inf2, Inf3, and Inf4). This network has become one of the prototypical systems for theoretical analyses of cell differentiation and morphogenesis [43], and it has been shown to be well-suited to explore new questions and propose new methodologies.

For example, recently an EL model for flower development based on a continues stochastic approximation of the Boolean GRN showed that characteristic multigene configurations emerge from the constraints imposed by the GRN; but the temporal pattern of cell transitions also seems to depend on the asymmetry in gene expression times-scales for some of the main regulators [33]. Based on this work, it was suggested that parameters representing finer regulatory processes, such as gene expression decay rates, enable richer and more accurate descriptions of the underlying cellular transitions. Specifically, the results suggested that relative differences in the decay rates of particular genes may be important for the establishment of the robust pattern of differentiation transition observed during floral organ determination. Thus, along with the constraints imposed by the GRN, a hierarchy of decay times of gene expression may define alternative routes to cell fates [21,33]. This possibility has not been studied systematically yet and it might prove crucial to undertand how such GRN modules are connected to signal transduction pathways that alter cell-fate attainment patterns.

Given the background exposed above, *a first question concerns the systematic exploration of the effect of a hierarchy of gene expression times on cell-fate specification during early flower development*. On the other hand, flower developmental mechanisms have been shown to result largely from the global self-organizational properties of the FOS-GRN; yet, it has not been straightforward to establish differences in the functional (dynamical) role of individual genes within the network. Therefore, *a second question concerns whether by analyzing gene dynamics we can test if there are such differences and, if so, if they correlate with biological observables*. Given that both questions require modeling exercises that go beyond a simple Boolean GRN model, in this contribution we first propose a modeling framework to extend the Boolean FOS-GRN model to a continuous system, and then show how it can be used to explore the questions addressedhere.

For the sake of concreteness, we frame the questions in the context of the dynamics of early flower development as follows: (1) We define the propensity of the Boolean stationary gene configuration to be transformed by changes of particular gene parameters as a proxy for gene functional role. (2) We test as a control parameter the genes characteristic decay rate in order to further explore the hypothesis raised in [33], that differences in gene decay rates may potentially guide cell-fate decisions during flower development. (3) We contrast the dynamical/biological classification with the known experimental data regarding the role of the ABC genes. In other words, we functionally classify the genes in the network by exploring their propensity to produce qualitative changes in the AL that would ultimately lead to cell-fate decisions (*i.e.,* attractor transitions). We also analyze the robustness of each attractor by means of their propensity (or lack thereof) to undergo such induced transitions. We hypothesize that there is a relationship between the impact of specific genes in the dynamics of the whole GRN, their biological function, and the observed hierarchy of differentiation events during early flower development.

Overall, this work constitutes a first step towards the dynamical, mechanistic characterization of the main molecular regulators of flower development; and provides a general methodological framework to approach similar questions in other developmental processes. It also provides hypotheses concerning which genes within the FOS-GRN are more likely to link this module to other regulatory circuits and signaling transduction pathways which might be crucial for the temporal progression of flower development. In conclusion, the approach put forward here allows analyses of the role of the genes’ decay rates in modifying the AL and thus affecting cell-fate transitions or patterning.

## Methods

### Modeling framework

**Figure 1:**
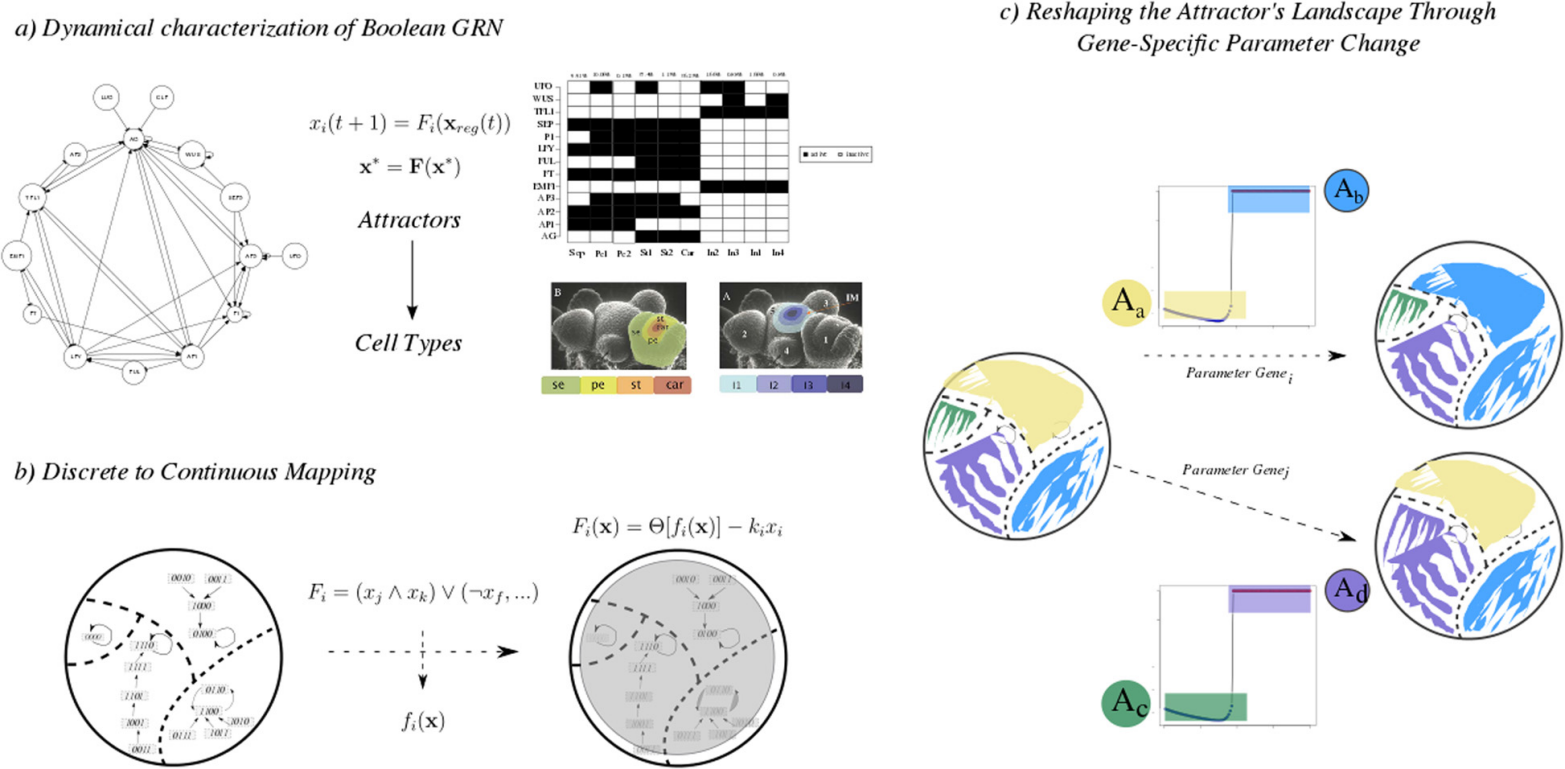
Schematic representations of the modeling methodology.**a)** The starting point is an experimentally grounded and dynamically characterized GRN Boolean model. Here the FOS-GRN is used, which recovers ten fixed-point attractors representing the cell-types observed during early flower development. **b)** The Boolean model is transformed into an equivalent continuous dynamical model. A set of rules is applied to the logical propositions of the Boolean model in order to derive a logic-based ODE model in continuous state-space. **c)** An attractor-wise, gene-wise numerical bifurcation analysis is performed. Because of qualitative changes to the AL induced by increasing parameter values several basins of attraction may merge into one, causing an inevitable cell-fate decision (*i.e.,* an attractor transition).

The scope of biological questions that Boolean GRN models are suited to address can be expanded. Here we focus on two specific questions that are important for developmental biology and which cannot be addressed by Boolean models – as originally proposed. (1) Although gene knockout or over-expression experiments are straightforward to simulate using a Boolean model, the richness of gene interactions may be more thoroughly explored by considering the intertwined dynamics of differentiation stimuli (microambient alterations, chemical signaling, catalytic reactions, etc.) and gene characteristic expression times which determine the developmental process itself, and which are not easily taken into account in a Boolean approach due to the absence of genes’ specific parameters. (2) It is not straightforward to study potential transition events among the already characterized stable cellular phenotypes with the Boolean deterministic formalism. With this limitations in mind, here we propose a novel modeling framework as an extension of the original Boolean GRN model. Our goal was to devise an extended methodology able to circumvent these limitations while maintaining the simplicity and clarity of the Boolean model. The proposed framework includes the following steps (see Figure 1): (1) the characterization of the dynamical behavior of an experimentally grounded Boolean GRN - and its associated AL, (2) the transformation of the Boolean model into a system of ordinary differential equations (ODEs) with an equivalent AL, (3) an attractor-wise, gene-wise numerical bifurcation analysis using the characteristic decay rate of each gene as a control parameter [43,44], and (4) the classification of genes into groups according to their propensity to induce qualitative changes to the AL and their potential to cause specific transitions between attractors.

#### Boolean GRN model

A Boolean network is a dynamical model with discrete time and discrete state variables. This can be expressed formally as:

(1) $$ x_{i}(t+1) = F_{i}(x_{1}(t),x_{2}(t),\ldots,x_{k}(t)),  $$

where the set of functions *F*_*i*_ are logical prepositions (or truth tables) expressing the relationship between a gene *i* and its *k* regulators, and where the state variables *x*_*i*_(*t*) can take the discrete values 1 or 0 indicating whether the gene *i* is expressed or not at a certain time *t*, respectively. An experimentally grounded Boolean GRN model is completely specified by the set of genes proposed to be involved in the process of interest and the associated set of logical functions derived from experimental data [21]. The set of logical functions for the FOS-GRN used in this study is included in Additional file 1. The dynamical analysis of the Boolean network model was conducted using the package *BoolNet* [45] within the *R* statistical programming environment (www.R-project.org).

#### Continuous GRN model

In order to characterize qualitative changes in the dynamics of the GRN under continuous variations of a given parameter (here a gene’s decay rate) we study a continuous representation of the discrete Boolean dynamics. Several approaches have been used to describe a Boolean GRN as a continuous system [21,33,46,47]. Here we adopt a system of ODEs of the form:

(2) $$  \frac{{dx}_{i}}{dt} = \Theta[f_{i}(x_{1},x_{2},\ldots,x_{k})] - k_{i}x_{i},  $$

where *k*_*i*_ represents the expression decay rate of the gene *i* of the GRN. The function *f*_*i*_ results from performing a transformation to the corresponding boolean function *F*_*i*_ following the rules:

(3) $$ \begin{aligned} & x_{i}(t) \, \wedge \, x_{j}(t) \, && \rightarrow \, x_{i}(t) \,. \, x_{j}(t), & \\ & x_{i}(t) \, \vee \, x_{j}(t) \, && \rightarrow \, x_{i}(t) \, + \, x_{j}(t) \, - \, x_{i}(t) \,. \, x_{j}(t), & \\ & \neg x_{i}(t) \, && \rightarrow \, 1 - x_{i}(t). & \end{aligned}  $$

Following [21,33] we consider that the input-response function associated to each gene displays a saturation behavior characterized by a logistic function. In this case, the input associated with the gene *i* takes the form:

(4) $$ {\small{\begin{aligned} \Theta[f_{i}(x_{1},x_{2},\ldots,x_{k})] = \frac{1}{1+\exp[-b[f_{i}(x_{1},x_{2},\ldots,x_{k})-\epsilon]]}, \end{aligned}}}  $$

where *ε* is a threshold level (usually *ε* = 1/2), and *b* the input saturation rate. For *b*>>1, the input function displays dichotomic behavior. A stationary state is defined by *dx*_*i*_/*dt*=0, so that Eq.(2) yields

(5) $$ {x_{i}^{s}}=\frac{1}{k_{i}} \Theta\left[f_{i}\left({x_{1}^{s}},{x_{2}^{s}},\ldots,{x_{k}^{s}}\right)\right],  $$

where ${x_{i}^{s}}$ denotes the stationary value. We observe that the expression level of the GRN node *i* is inversely proportional to its decay rate, so that for a fast decay rate *k*_*i*_≫1 the expression level ${x_{i}^{s}} \to 0$, while for a slow decay *k*_*i*_≪1, ${x_{i}^{s}} \gg 1$. Thus, a hierarchy in gene decay rates determines a pattern of relative gene expression levels.

The obtained system of ODEs is included on Additional file 1. Similar logic-based ODE models have been presented before (see, for example [48,49]). The numerical analysis of the system of ODEs was conducted using inhouse *R* code exploiting the functions provided in the packages *deSolve* [50] and *rootSolve* [50], as described in [51]. During preliminary simulation experiments we observed that under the specified parameter values the uncovered fixed-point attractors always showed extreme values – *i.e.,* close to either 0 or 1, but not to 0.5.

#### Attractors landscape operational definition

The Attractors Landscape (AL) is specified by the exhaustive characterization of the state-space. We operationally define the AL as the data structure containing two elements: (1) a 2^*n*^×*n* state-space matrix, a matrix whose rows correspond to each of the 2^*n*^ possible states of a Boolean GRN; and (2) a vector of length 2^*n*^ whose elements take values *A*_*i*_ from the set {1,…,*A*_*n*_} where *A*_*n*_ is the number of attractors of a given Boolean Network. This structure thus maps each state to its corresponding attractor. For the case of the ODEs model, the obtained attractor states were discretized in order to have a direct comparison with the Boolean model. Following [52] an unsupervised k-means clustering algorithm [53] with two clusters (*i.e.,k*=2) corresponding to the two binary values was used for the discretization task (for details see [52]).

#### Bifurcation analysis

All bifurcation analyses were conducted numerically using the following algorithm. A specific attractor is taken as an initial condition in an ODEs initial-value problem. For each active gene in the attractor state: (1) an ordered set of values for the control parameter (here the gene’s decay rate *k*_*i*_) is chosen – while the rest of the parameters are kept constant; (2) the ODEs are solved numerically until reaching an steady state, each time using a different parameter value, and for all the parameter values in the set; and (3) a plot is generated with parameter values in the x-axis and the total sum *y* of the single gene expression values for the *n* genes (*i.e.,*$y=\sum _{i=1}^{n} x_{i}^{*}$) of the obtained steady state $x_{i}^{*}$ in the y-axis. The analysis is performed for each attractor. Qualitative changes are identified by the occurrence of sudden jumps in the bifurcation graphs.

### Data analysis

#### Network topology

For each gene (node) in the FOS-GRN the following measures of topological importance were calculated: degree (number of nodes it is connected to), in-degree (number of connections directed towards it), out-degree (number of connections directed towards other nodes), and betweenness (fraction of all shortest paths that pass through it). All network topological computations were conducted using the *igraph* package [54]. In order to test for the association of the genes propensity to produce AL qualitative changes and their topological features within the network, simple linear regression models were fitted using the calculated propensity of each gene to produce a qualitative change as response variable and each topological feature as predictor.

To test whether interacting genes in the FOS-GRN have a related propensity to produce AL alterations in response to an increase in their decay rate. The average absolute difference of the value of the calculated gene sensitivity between interacting components in the network was calculated and then used as a statistic in a simulation (sampling) procedure in order to assess how frequently it is expected to observe this or a smaller value in an ensemble of similar but random networks. Specifically, 100,000 networks each with the same number of nodes and interactions were generated, and the statistic was calculated for each of these networks. The estimated distribution of the statistic over the ensemble of networks was then used to calculate the probability of observing a value equal or smaller than that calculated in the FOS-GRN.

## Results

### Dynamical analysis of the GRN

The GRN underlying early flower development (refered to as FOS-GRN) was used as a study case. The most recent version reported in [33] was used. The corresponding logical update rules are reported in Additional file 1. The first task was to characterize the GRN dynamical behavior and its associated AL. The global dynamical behavior of the network was analyzed by the exhaustive characterization of all steady states using all possible initial conditions. Specifically, we calculated its attractor states and their corresponding basins of attraction. We arranged both initial conditions and corresponding attractor into an AL structure (see methods). As expected, the network recovered 10 fixed-point attractors: four corresponding to the four regions of the inflorescence meristem (Inf1, Inf2, Inf3, and Inf4), and six to the four floral organ primordial cells within the flower primordia (Sep, Pt1, Pt2, St1, St2, and Car). The two attractors corresponding to petals (Pt1 and Pt2) are identical except for the state of activation of the UFO gene, and the same holds for the two stamen attractors (St1 and St2). The attractors and its basins are reported in Additional file 1. We then transformed the Boolean network into a system of ODEs (see Methods).

A series of studies have extensively validated the Boolean FOS-GRN model in terms of increasingly available experimental data; for example, it has been shown that its dynamical behavior is robust enough as to predict the experimentally induced phenotypes in several mutant conditions [11,19,24,55]. In order to preserve such validated behavior we derived a ODEs model preserving the attractors and basins of attraction uncovered in the Boolean case. The input-response function included in the proposed continuous model contains 2 parameters: *b*, and *ε*. The value of the parameter *b* was chosen as the smallest integer value able to recover the same fixed-point attractors and their basins of the Boolean model. We tested a range of values *b*=*i* for [1,..*i*..,40]. We found that a value of *b*≥5 is able to recover the same attractors and basin sizes that the ones uncovered with the Boolean model. We use a value of *b*=5 for all the following calculations. The *ε* parameter is a threshold level, for simplicity a value of *ε*=0.5 was used. For this first analysis the decay parameter for each gene was set to *k*_*i*_=1. The 10 attractors obtained with these settings, and its basins size are shown in Additional file 1. Thus, we derived two dynamical models for the FOS-GRN with an equivalent behavior in terms of the uncovered attractors and basins of attraction. We specified an AL structure for each model.

### Bifurcation analysis

**Figure 2:**
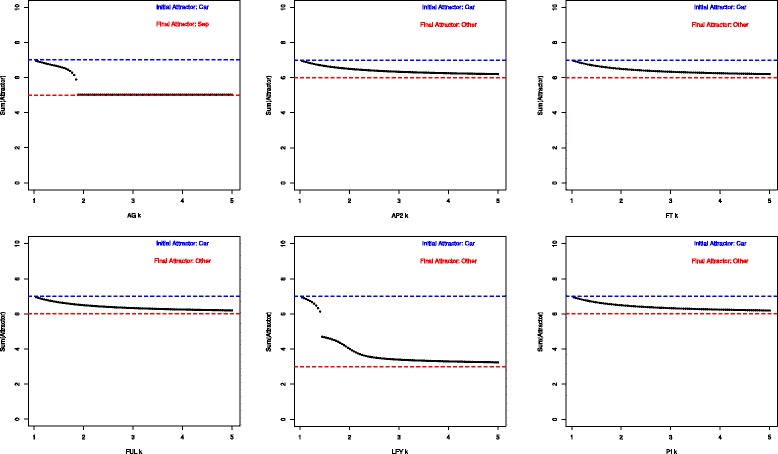
Bifurcation diagrams. Graphs obtained as a result of the Bifurcation analysis performed for the genes corresponding to carpels (Car) attractor. The genes AG and LFY were able to induce qualitative changes. The gene AG produced a transition between already characterized attractor states (Car → Sep). The change in LFY produced a new attractor state.

We performed a numerical analysis in order to explore the propensity of single genes to qualitatively change the attractor states where they are expressed (and thus induce attractor transitions in the AL) in response to an increase in their decay rate parameter (see Methods). To illustrate our analyses, we generated a set of graphs, one per each gene expressed in each attractor. In the graph we plotted the initial attractor state and its progressive change resulting from altering the decay parameter *k*_*i*_. If *m* genes were active in the attractor in question, the analysis was conducted for each gene *i* for *i*=[1,…,*m*]. We performed the analysis to each attractor *j* for *j*=[1,…,10]. Figure 2 shows the graphs obtained for the genes corresponding to carpels (Car) attractor. In this case, only the genes AG and LFY were able to induce an phase transition. Whereas gene AG produces a transition between already characterized attractor states (*Car* →*Sep*), the change in LFY produces a new attractor state. The graphs for all the attractors (and their genes) are reported in Additional file 2. We found that for each attractor at least one of its expressed genes is able to produce a qualitative change to the AL. Some genes (attractors) are more likely to produce (undergo) attractor transitions. These results suggest that, by systematically testing the potential of altering specific genes qualitatively changes the GRN underlying AL, we can uncover differences in the genes functional (dynamical) role in the overall system underanalysis.

### Gene classes

**Figure 3:**
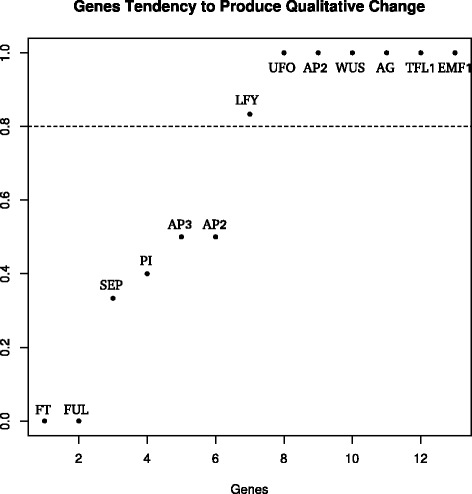
*PT* metric values. The plot shows propensity to induce phase transitions quantified for each gene. The horizontal line divides the genes into groups of higher (above) of lower (below) propensity. The two groups are based on a clustering analysis.

**Figure 4:**
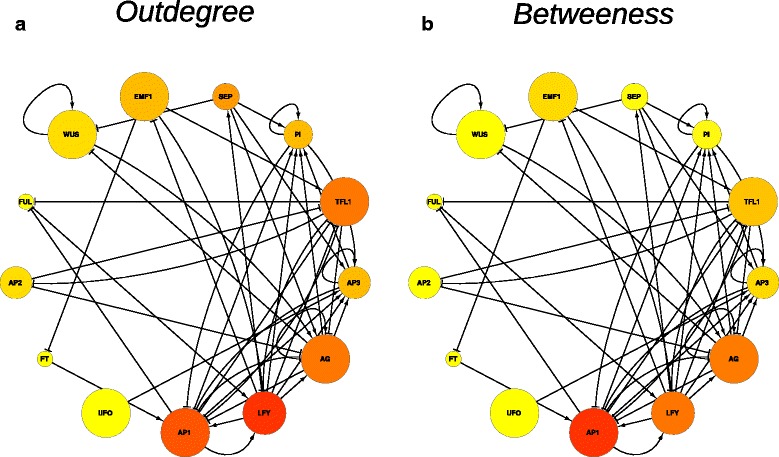
The FOS Gene regulatory network. The graph represents the mapping of the calculated *PT* values with the topological features out-degree **(a)** and betweenness **(b)** into the graph of the FOS-GRN. The size of the nodes represents the *PT* values. The topological features are represented by a graded yellow-red color scale with yellow (red) in the left (right) extreme.

In order to have a better understanding of the nature of the uncovered differential functional (dynamical) role of genes, we classified the genes according to their propensity to induce attractor transitions. Table A1, in Additional file 1 summarizes the result of all the bifurcation analyses. For each attractor, and for each perturbed gene, we registered whether a qualitative change is produced or not, and the final attractor attained after the simulated change. In order to numerically express the propensity of each gene to induce qualitative changes, we counted the number of times a gene is able to produce a qualitative change and normalized this number by the number of times the gene is expressed among the 10 attractors. The resulting scale is shown in Figure 3. We will refer to this quantified propensity to induce qualitative changes (*phase transitions*) as the metric *PT*. In order to classify a gene with either high or low propensity, we clustered the genes described by the quantified propensity *PT* in two groups using the k-means clustering algorithm [56]. According to this analysis, the genes with higher propensity are: UFO, AP1, WUS, AG, TFL1, EMF1, and LFY (see Figure 3). On the other hand, genes were also classified depending on whether or not, when they induce a qualitative change, are able to induce a transition between already characterized attractor states. The genes found to be able to produce this type of transitions are: UFO, AP1, WUS, AP3, AG, TFL1, EMF1, and PI. Additionally, we also classified the genes depending on whether or not they are able to produce new attractor states after the qualitative change. The genes that show this behavior are: SEP, AP2, PI, LFY. The three classes are shown in Table 1. In Figure 4 we map to each node in the graph of the GRN its corresponding metric *PT*.

**Table 1:** **Gene classes according to their propensity to produce qualitative changes to the attractors**

**Classification**	**Genes**
High propensity genes	UFO, AP1, WUS, AG, TFL1, EMF1, LFY
Low propensity genes	SEP, FT, AP3, AP2, PI, FUL
Genes causing transition	UFO, AP1, WUS, AP3, AG, TFL1, EMF1, PI
between known attractors	
Genes causing transition	SEP, AP2, PI, LFY
between unknown attractors

### Analysis of the classes of genes

**Figure 5:**
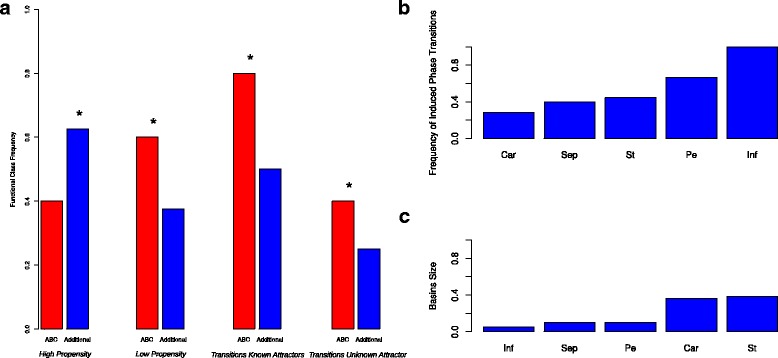
Genes propensity and functional class.**a)** The plot shows the gene frequency of each functional group (i.e., ABC, or Additional) within each gene class (i.e., High Propensity Genes, Low Propensity Genes, Genes Causing Transition Between Known Attractors, Genes Causing Transition Between Unknown Attractors). The star sign represents gene group over-representation as defined by a lower p-value relative to the other gene functional class calculated with a hypergeometric test (see Methods). **b)** The plot shows the calculated attractors propensity to undergo attractor transitions. **c)** Attractors basin size plot.

In order to test if there is evidence of an association between the differential functional role of genes and background biological knowledge, we compared the representation of the ABC genes and the additional (non-ABC) genes of the FOS-GRN within each of the classes described in the previous subsection, and listed these in Table 1. We followed two procedures: (1) calculated the gene frequency of each biological group (e.g. ABC, or Additional) within each gene class, (2) perform a hypergeometric test for biological group over-representation. Figure 5a shows the results. We found the following patterns. In the classes defined by the gene propensity to induce qualitative changes, there is a lower (higher) representation of ABC genes in the high (low) propensity class with respect to the other additional genes. On the other hand, in the classes defined by the gene capacity to produce attractor transitions between known or unknown attractors, there is a higher representation of ABC genes with respect to the other additional genes in both classes. These results suggest that ABC genes are less likely to produce qualitative changes in the AL by induced changes in their expression dynamics - at least under a relatively higher decay rate as tested here - than the non-ABC genes in the network. On the other hand, if such a qualitative change occurs, ABC genes are more likely to both induce inter-attractor transitions and to specify novel attractors than the non-ABC genes in the network. These seemingly contradictory results can be understood by taking into consideration the relative robustness of the different attractors against such parameter perturbations (see below).

### Attractors propensity to undergo transitions

Taking in consideration that not all the genes are expressed in all the attractors, we also compared the propensity of the different attractors to undergo attractor transitions by calculating the frequency of attractor transitions per attractor as the number of undergone attractor transitions normalized by the number of genes expressed in the respective attractor. The results are shown in Figure 5b. For this analysis we mapped all the states in the AL corresponding to any of the four inflorescence attractors (Inf1, Inf2, Inf3, and Inf4) into a single *Inf* attractor. We also mapped the states of the attractors (St1, St2) and (Pt1, Pt2) to the individual attractors *St* and *Pt*, respectively. Hence, the system had a total of five attractors. We found that the inflorescence attractor is the attractor with the highest propensity. Specifically, a relatively higher decay rate of any of the genes expressed in the inflorescence attractors (TFL1, EMF1, UFO, WUS) with respect to the other genes always produces an attractor transition. Three of the flower attractors (Car, Sep, St) show a frequency of attractor transitions lower than 0.5, while the remaining flower attractor (Pe) shows a frequency ∼0.6. These results suggest a relationship between the propensity of undergoing attractor transitions and the direction of differentiation during early flower development - being less likely to induce attractor transitions as the course of development progresses, or to produce a reprogramming from a floral organ attractor to an inflorescence one. Interestingly, attractors propensity to undergo attractor transitions do not correlate with the attractors basin sizes (see Figure 5b), as intuitively expected.

### Genes propensity to produce qualitative changes and network structure

Given that it is common to provide evidence of the gene importance in the context of networks by considering only each gene’s topological features [57], we tested if the gene’s propensity to produce qualitative changes to the AL as defined here is correlated with topological properties. Specifically, we tested an association between each of genes topological features and the quantified gene’s propensity of producing a qualitative change to the AL (*PT* metric) by performing linear regression analyses. We characterized each node by a set of network topological features, which express numerically the placement of each gene within the network. For each gene (node) in the FOS-GRN we calculated two commonly used measures of topological importance: degree (number of nodes it is connected to), and betweenness (fraction of all shortest paths that pass through it). We also considered that the dynamical behavior of the GRN is associated with the type of interactions within the network, thus we specified further the degree feature into in-degree or out-degree. Interestingly, we found a significant relationship between *PT* metric and two predictor variables: out-degree and betweenness (p-value = 0.03). In Figure 4 we represent graphically the associations by mapping the *PT* values and the topological features out-degree (Figure 4a) and betweenness (Figure 4b) into the graph of the FOS-GRN. The size of the nodes represents the *PT* values in the scale [0,…,1]. The topological feature is represented by a graded yellow-red color scale with yellow (red) in the left (right) extreme [0,…,1].

#### Similarity in the propensity of interacting genes

To further test if there is an association between gene’s topological features and their propensity to produce qualitative changes in the attractors, we performed the following analysis. Given the *PT* values for each gene, we asked if interacting genes within the FOS-GRN share more similar propensity within themselves than with non-interacting components. This pattern, if found, would suggest a close relationship between network architecture and such gene’s dynamical property. Similar analyses have been proposed in network-based molecular evolutionary studies as a test for an association between network structure and evolutionary constraint [58,59]. In order to test whether this pattern is present in the FOS-GRN we calculated the average absolute difference (AAD) of the *PT* value between interacting components in the networks and used it as an statistic. An AAD of *PT* of 0.333 was calculated for the FOS-GRN. We then tested how likely is this value to be explained by change alone; specifically, we generated a null distribution by calculating AAD values in an ensemble of similar but random networks. We include the histogram of the corresponding statistic on an ensemble of 100,000 random networks with the same number of nodes and interactions in Additional file 1. Based on this data we estimated the probability of observing such a small value by calculating the fraction of random networks showing an AAD value *AAD* ≤0.333 or greater. The resulting probability was 0.06.

Taken together these results: (1) a significant relationship between *PT* metric and the topological features of out-degree and betweeness, and (2) a marginally significant (p-value ∼0.06) similar propensity within interacting genes; support the hypothesis that there is a close relationship between a gene’s placement in the network, or its micro-topological position within a GRN, and its propensity to produce qualitative changes to the AL – at least in the case of the FOS-GRN. More general analyses for GRN with different topologies and architectures should be done.

## Discussion

Recently, several authors have considered the restructuring of the state-space associated with a dynamical model of a GRN as an important aid for understanding underlying mechanisms occurring during development an evolution [5,32,60-65]. A conclusion is emerging: the model of a landscape changing over time seems plausible as an explanation for fundamental features of morphogenesis and tissue formation [13]. In general, however, most work in this regard has been centered around either conceptual discussions or the dynamical analyses of small gene circuits. The exploration of such questions in larger, multi-attractor GRNs, that are grounded on experimental data and underlie realistic cases of cell differentiation, and in which the state-space presents a more complex structure, has largely been left behind. Here we present a modeling framework of general applicability as a first step for such type of exploration. For the sake of concreteness, we used as a model GRN the specific case of the FOS-GRN.

ODE-based models allows more flexible choice of network parameters reflecting, for example, different interaction strengths or inductive signals. Analyses of mathematical models of differentiation dynamics have shown that the considerations of such flexibility may be important to understand and control cell-fate choices (see, for example [5,9]). In the present case, given the hypothesis raised by some of the authors in [33] that differences in gene decay rates may potentially guide cell-fate decisions during flower development; we focus exclusively on the impact of relative gene decay rates in restructuring the AL, and thus we limit the scope of our conclusions. Additionally, the specific biological mechanisms driving such differential expression dynamics in vivo are not known. We speculate that signaling modules regulating responses to environmental cues may be directly connected to some of the components included in the GRN module analyzed here. In this direction, some of the authors have recently started to characterized such integrated GRNs considering the relevance of light sensing in flowering developmental choices [66]. Future work will test the effect of coupling such signaling modules with the GRN analyzed herein on the structure of the AL.

In the present case, when a given gene’s decay rate is tuned and crosses a threshold, we observe qualitative changes in the AL’s organization. We refer to the different patterns of organization as *phases*. The study of complex systems is, to a large extent, a search for the principles pervading self-organized, emergent phenomena and defining its potential phases [43,67]. Following this complex systems perspective, in this work we thus explored the phase changes in the AL that emerge from the dynamics of an experimentally grounded, complex GRN. Such transition phenomena are collective by nature and result from interactions taking place among the interacting genes of the GRN and not by any single gene alone. In any case, our exploration helped uncover a differential role of individual genes regarding their propensity to produce these induced *phase transitions*.

Given that the observed phase changes effectively correspond to qualitative changes of the AL in which one or more of the attractors (cell states) disappear, the result would inevitably lead to an induced cell-fate decision. We focus on these latter attractors transitions. We must point out that in the present case we study the induced qualitative changes of the AL indirectly by systematically analyzing the local effects on each attractor of quantitative changes in gene decay rates. The relative stability of each attractor’s basin is expected to be relevant in constraining transitions among attractors. This latter problem is the subject of current intense research and is more naturally approached by using stochastic models (see, for example [34,68]).

Differences in decay rates may also be interpreted as different time-scale regimes. Interestingly, a recent study stressed the relevance of time delays arising from multistep chemical reactions or cellular shape transformations [69]. Specifically, the authors argue in this reference that such feature is crucial in understanding cell differentiation, as it leads to novel states in epigenetic landscapes. In the present case, we indeed found that relatively different gene time-scale regimes produce qualitative changes to the otherwise static AL. Unlike the generic model presented by Mitra and collaborators [69], however, here we studied the dynamical behavior of specific genes which have been extensively characterized experimentally during decades of plant developmental genetics studies (see, for example [2]).

Most studies on the molecular basis of floral development focus on the eukaryotic MADS-box gene family, particularly floral homeotic genes such as AGAMOUS (AG), APETALA3 (AP3), PISTILLATA (PI), and several AGAMOUS-like genes [70]. Such genes are also the most important constituents of the ABC model for flower organ specification described above. Although based on extensive experimentation, the ABC genes have been characterized as having a prominent, functional role in cell fate and organ type specification during early flower development yielding homeotic transformations among floral organ when mutated; it was only a mechanistic view, the FOS-GRN dynamical model, which provided a sufficient explanation for the empirically observed ABC patterns – i.e., the combinatorial ABC code and the stable gene expression configurations observed during early flower development in *Arabidopsis* [2,11,19]. This model has been studied from different perspectives [24,33,41].

When testing the coherence of experimental data regarding the role of these molecular regulators under the framework of a GRN dynamical model certain questions arise. Why the ABC genes and not the other genes in the network display *homeotic* mutations when they are inactivated? Is there a relationship with this characterized biological (functional) property and its dynamical behavior within the FOS-GRN? What genes are more prone to have a stronger influence on the dynamical behavior of the whole system, and thus the phenotype, when perturbed or coupled with other circuits, signaling mechanisms, or processes outside the GRN module? Here we present a methodological framework for systematically testing the potential of specific genes when perturbed to produce qualitative changes to the underlying AL. By applying this approach to the FOS-GRN we uncover differences in the functional (dynamical) role of their genes. We speculate that such dynamical behavior might give information about which genes are most likely to be links with other circuits and processes.

A somewhat unexpected result is that the homeotic genes are less likely to produce attractor transitions in the AL by an induced higher decay rate, in comparison to other non-ABC genes in the network (see Methods). However, if we consider that ABC genes specify floral organ identity, a late process in early flower development, a higher robustness to non genetic perturbations such as changes in gene expression parameters is consistent with an increased stability of the cellular phenotypes as development proceeds. Indeed, when analyzing the propensity of the different attractors to undergo attractor transitions (see Methods) we found that the attractors corresponding to the flower cell-types show a lower propensity that the Inflorescence attractors (see below). On the other hand, we also found that in the cases where a phase transition induced by higher decay rates of ABC genes relative to the rates of other genes, the output is more likely to produce both an induced inter-attractor transition and to specify a novel attractor. This result aligns well with the empirical status of the ABC genes as *homeotic* genes, as it suggests that higher enough perturbations slowing gene function that approach a loss-of-function mutation, eliminate or produce specific cellular phenotypes, that correspond to changes of attractors, and thus homeotic alterations.

In Alvarez-Buylla and collaborators [24] some of the authors proposed a mechanistic explanation for the stereotypical temporal pattern of cell-fate specification during early flower development by means of noise-induced attractors transitions. In that study, however, it was shown that stochasticity alone was not able to explain a transition from the inflorescence to the flower meristems (attractors), an early, well-characterized event during flower development. Thus the authors speculate on the role of non-random inductive signals in the transition from cell fates in the inflorescence meristem to those in the flower meristem [24]. Our results suggest that this indeed could be the case, as a relatively higher decay rate of any of the genes expressed in the inflorescence attractors (TFL1, EMF1, UFO,WUS), with respect to the other genes, always produces a phase transition, and this transitions predominantly lead to flower organ attractors (see results). Thus, our model uncovered a potential mechanism which could be subjected to experimental validation. Namely, TFL1, EMF1, UFO, or WUS genes have a relatively higher gene decay rate relative to flower specification genes during early flower development and within the inflorescence meristem. This feature in turn facilitates the inflorescence-flower transition when these genes are altered in their decay rates, thus suggesting that signals or pathways at play during the transition from inflorescence to flower meristem should interact or affect decay rates of these genes. In contrast, most functional studies concerning inflorescence to flower transition, have mostly focused on LFY and also on AP1 [71,72].

The distinction between molecular network structure and function is a core problem in systems biology. Dynamical GRN models enable a rigorous distinction between structure (topology) and function (dynamics). In a recent molecular evolutionary study also using the FOS-GRN, it was suggested that the dynamical functional role of genes within the network, and not just its connectivity, could play an important role in constraining evolution [59]. Such hypothesis implies a close relationship between network structure and function. Based on our operational definition of the gene functional role as the gene’s propensity to produce AL attractor transitions, we asked if this property is associated with the gene’s network topological features. We found that a significant correlation among these two. Our results thus support the hypothesis that for the FOS-GRN there is a close relationship between a gene’s placement in the network and its propensity to produce attractor transitions in the AL. Likewise our results also provide partial support for the dynamical functional role of genes being important for constraining evolutionary changes.

## Conclusions

In this contribution we present a methodology of general applicability as a first step for exploring the restructuring of the state-space associated with a dynamical multi-attractor GRN model. The framework consists on systematically exploring the propensity of single genes to produce qualitative changes in the AL as a result of changes in their parameters. Importantly, different GRN models and the effect of general inductive signals can be explored within the same framework. We showed how biological insights can be derived by applying the methodological framework to a single well-characterized and experimentally grounded GRN: the FOS-GRN. Future studies should explore if the results derived for this GRN can be generalized to GRN with contrasting typologies and architectures.

We systematically explored the effect of relative differences in gene decay rates on AL structure, and showed that by analyzing gene dynamics we can test if there are differences in the functional (dynamical) role among individual genes within the network, and that such differences correlate with biological observables. Specifically, (1) the dynamical behavior of ABC genes provide both robustness and flexibility in response to parameter perturbations, and are prone to both produce inter-attractor transitions and specify novel attractors; (2) It is less likely to induce attractor transitions as the course of development progresses; (3) non-random inductive signals may be at play in the transition from cell fates in the inflorescence meristem to those in the flower meristem; and (4) for the FOS-GRN there is a close relationship between a gene’s placement in the network and its dynamical role. Taking together, our results suggest that there is a relationship between the impact of specific genes in the dynamics of the whole FOS-GRN, their biological function, and the observed hierarchy of differentiation events during early flower development.

## Additional files

Additional file 1
**Supporting Figures and Tables.** The Additional file 1 includes the following information: Figure A1. Boolean FOS-GRN logical update rules. Figure A2. Attractors of the Wild-type Boolean FOS-GRN. Figure A3. ODEs model of the FOS-GRN. Figure A4. Attractors of the Wild-type ODEs FOS-GRN Model. Figure A5. Comparison of the Attractors and Basins Uncovered with the Boolean and ODEs FOS-GRN Models. Table A1. Table summarizing the result of all the bifurcation analyses. Figure A6. Histogram of the average absolute difference in PT values calculated from simulated networks values.

Additional file 2
**Results of the Bifurcation (Phase transition) Analysis.**
