# Supplementary material for: Reshaping the epigenetic landscape during early flower development: induction of attractor transitions by relative differences in gene decay rates
Source: BMC Syst Biol. 2015 May 13;9:20. doi: 10.1186/s12918-015-0166-y (PMC4438470; doi:10.1186/s12918-015-0166-y)
Supplement: Additional file 1 — Supporting Figures and Tables. The Additional file 1 includes the following information: Figure A1. Boolean FOS-GRN logical update rules. Figure A2. Attractors of the Wild-type Boolean FOS-GRN. Figure A3. ODEs model of the FOS-GRN. Figure A4. Attractors of the Wild-type ODEs FOS-GRN Model. Figure A5. Comparison of the Attractors and Basins Uncovered with the Boolean and ODEs FOS-GRN Models. Table A1. Table summarizing the result of all the bifurcation analyses. Figure A6. Histogram of the average absolute difference in PT values calculated from simulated networks values. [file 12918_2015_166_MOESM1_ESM.pdf]

Additional File 1

Figure A1. Boolean FOS-GRN logical update rules. The figure shows the model used in the study.

Figure A2. Attractors of the Wild-type Boolean FOS-GRN. Graphical representation of the 10 point attractors recovered by the dynamical analysis of the Boolean GRN model. Percentages above each column represent the corresponding attractor's basin size.

Figure A3. ODEs model of the FOS-GRN. Set of equations obtained after transforming the Boolean GRN model.

Figure A4. Attractors of the Wild-type ODEs FOS-GRN Model. Graphical representation of the 10 point attractors recovered by the dynamical analysis of the Boolean GRN model.

Figure A5. Comparison of the Attractors and Basins Uncovered with the Boolean and ODEs FOS-GRN Models.

Table A1. Table summarizing the result of all the bifurcation analyses. The columns give the following information: Column 1, initial attractors; Column 2, perturbed gene, Column 3, Binay variables indicating wheter a phase transition occurred (1) or not (0); Column 4, Attractor reached after the phase transition (NA when there was no transition, Other corresponds to a novel attractor).

Figure A6. Histogram of the average absolute difference in PT values calculated from simulated networks values. The graph shows the distribution of the average absolute difference in PT values calculated *on an ensemble of 100,000 random networks with the same number of nodes and interactions. The red vertical line indicates the corresponding value for the "real" FOS-GRN model.*

$x_i(t+1)$		$F_i(\mathbf{x}(t))$
$AG(t+1)$	$=$	$(\neg EMF1 \wedge \neg AP2 \wedge \neg TFL1) \vee (\neg EMF1 \wedge \neg AP1 \wedge LFY)$ $\vee(\neg EMF1 \wedge \neg AP2 \wedge LFY) \vee (\neg EMF1 \wedge \neg TFL1 \wedge LFY \wedge (AG \wedge SEP))$ $\vee(\neg EMF1 \wedge (LFY \wedge WUS))$
$AP1(t+1)$	$=$	$(\neg AG \wedge \neg TFL1) \vee (FT \wedge LFY \wedge \neg AG) \vee (FT \wedge \neg AG \wedge \neg PI)$ $\vee(LFY \wedge \neg AG \wedge \neg PI) \vee (FT \wedge \neg AG \wedge \neg AP3)$ $\vee(LFY \wedge \neg AG \wedge \neg AP3)$
$AP2(t+1)$	$=$	$\neg TFL1$
$AP3(t+1)$	$=$	$(LFY \wedge UFO) \vee (PI \wedge SEP \wedge AP3 \wedge (AG \vee AP1))$
$EMF1(t+1)$	$=$	$\neg LFY$
$FT(t+1)$	$=$	$\neg EMF1$
$FUL(t+1)$	$=$	$\neg AP1 \wedge \neg TFL1$
$LFY(t+1)$	$=$	$\neg EMF1 \vee \neg TFL1$
$PI(t+1)$	$=$	$(LFY \wedge (AG \vee AP3)) \vee (PI \wedge SEP \wedge AP3 \wedge (AG \vee AP1))$
$SEP(t+1)$	$=$	LFY
$TFL1(t+1)$	$=$	$\neg AP1 \wedge (EMF1 \wedge \neg LFY)$
$WUS(t+1)$	$=$	$WUS \wedge (\neg AG \vee \neg SEP)$

Figure A1. Boolean FOS-GRN logical update rules.

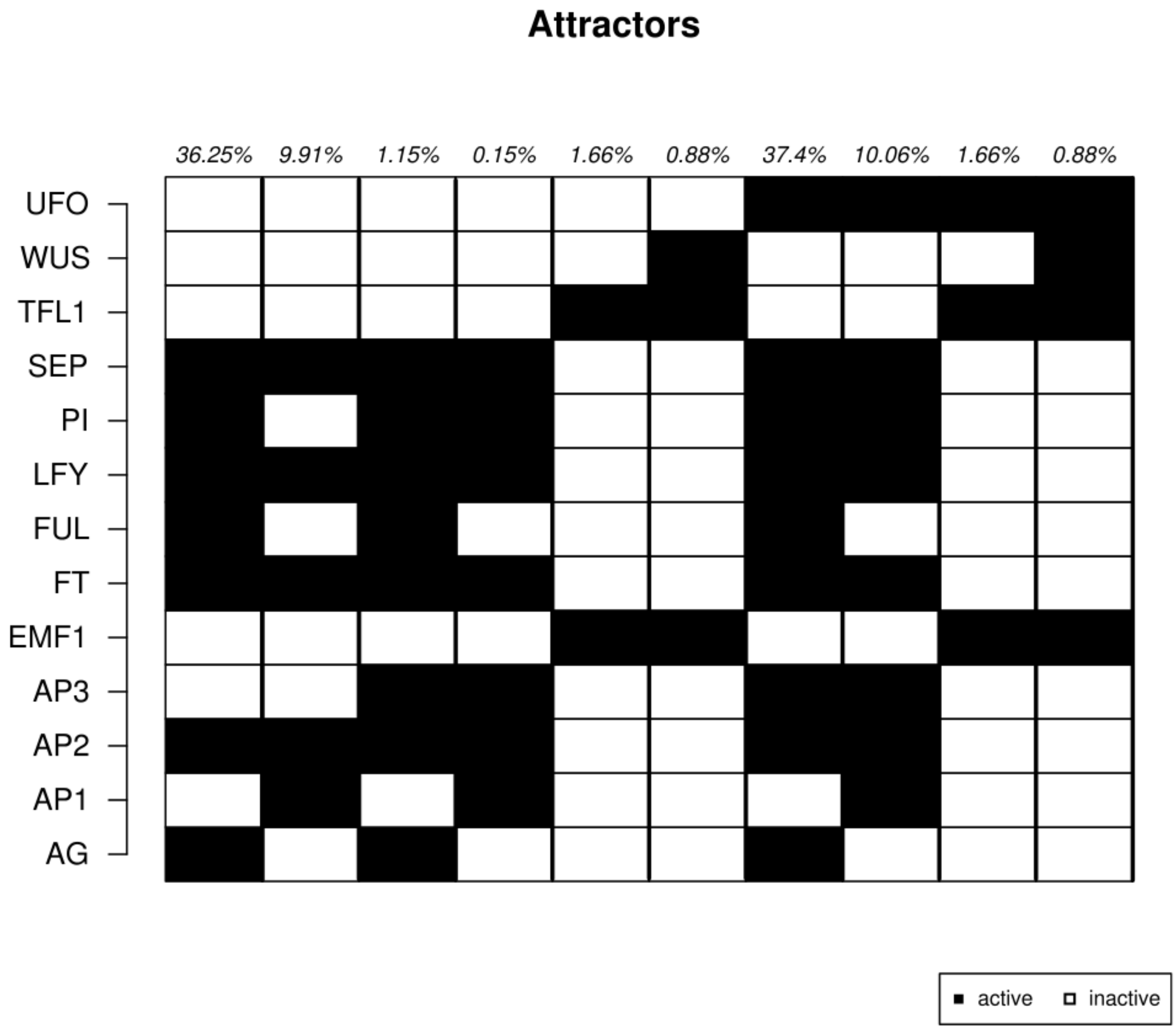

Figure A2. Attractors of the Wild-type Boolean FOS-GRN .

The ODEs model of the FOS-GRN takes the form:

$$\frac{dx_i}{dt} = \frac{1}{1+\exp[-b[f_i(x_1, x_2, ..., x_k) - \epsilon]]} - k_i x_i$$

where:

$$\begin{aligned}
f_{AG}(\mathbf{x}) &= LFY \cdot (1 - EMF1) \cdot (1 - ((AP1 \cdot AP2 \cdot (1 - WUS)) \cdot (1 - AG \cdot SEP \cdot (1 - TFL1)))) \\
&+ (1 - EMF1) \cdot (1 - TFL1) \cdot (1 - AP2) \\
&- (LFY \cdot (1 - EMF1) \cdot (1 - ((AP1 \cdot AP2 \cdot (1 - WUS)) \\
&\cdot (1 - AG \cdot SEP \cdot (1 - TFL1))))) \cdot ((1 - EMF1) \cdot (1 - TFL1) \cdot (1 - AP2)), \\
f_{AP1}(\mathbf{x}) &= (1 - AG) \cdot (1 - TFL1 \cdot (1 - LFY * FT)), \\
f_{FUL}(\mathbf{x}) &= (1 - AP1) \cdot (1 - TFL1), \\
f_{FT}(\mathbf{x}) &= 1 - EMF1, \\
f_{EMF1}(\mathbf{x}) &= 1 - LFY, \\
f_{LFY}(\mathbf{x}) &= 1 - EMF1 \cdot TFL1, \\
f_{AP2}(\mathbf{x}) &= 1 - TFL1, \\
f_{WUS}(\mathbf{x}) &= WUS \cdot (1 - AG \cdot SEP), \\
f_{SEP}(\mathbf{x}) &= LFY, \\
f_{PI}(\mathbf{x}) &= (LFY \cdot (AG + AP3 - AG \cdot AP3)) \\
&+ (PI \cdot SEP \cdot AP3 \cdot (AG + AP1 - AG \cdot AP1)) \\
&- (LFY \cdot (AG + AP3 - AG \cdot AP3)) \cdot (PI \cdot SEP \cdot AP3 \cdot (AG + AP1 - AG \cdot AP1)), \\
f_{AP3}(\mathbf{x}) &= (LFY \cdot UFO) + (PI \cdot SEP \cdot AP3 \cdot (AG + AP1 - AG \cdot AP1)) \\
&- (LFY \cdot UFO) \cdot (PI \cdot SEP \cdot AP3 \cdot (AG + AP1 - AG \cdot AP1)), \\
f_{TFL1}(\mathbf{x}) &= (1 - AP1) \cdot (1 - LFY) \cdot EMF1.
\end{aligned}$$

Figure A3. The ODEs model of the FOS-GRN

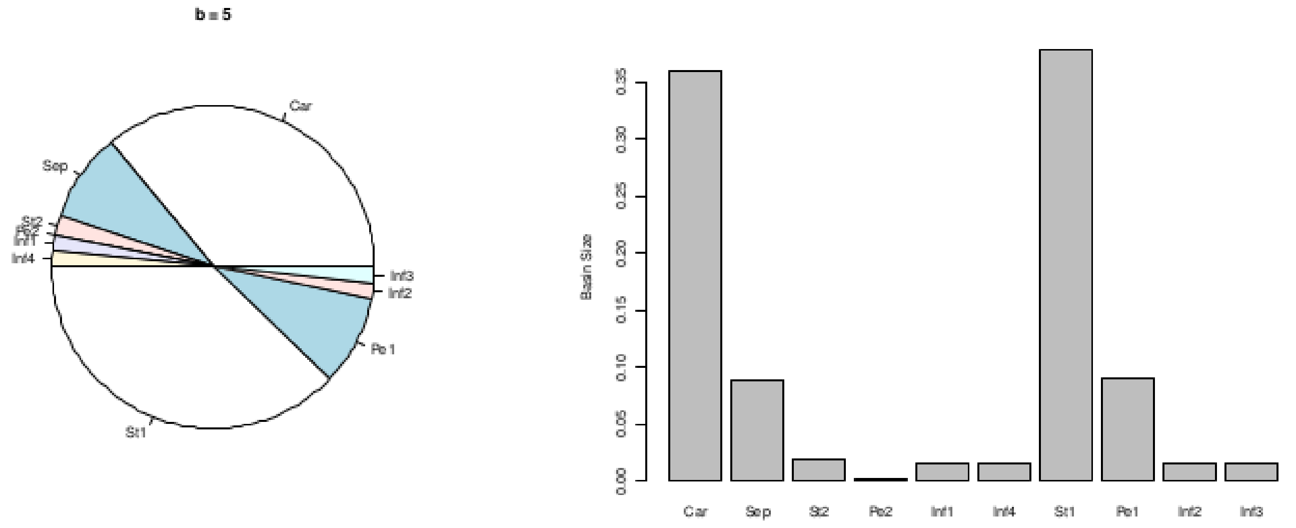

Figure A4. Attractors of the Wild-type ODEs FOS-GRN Model.

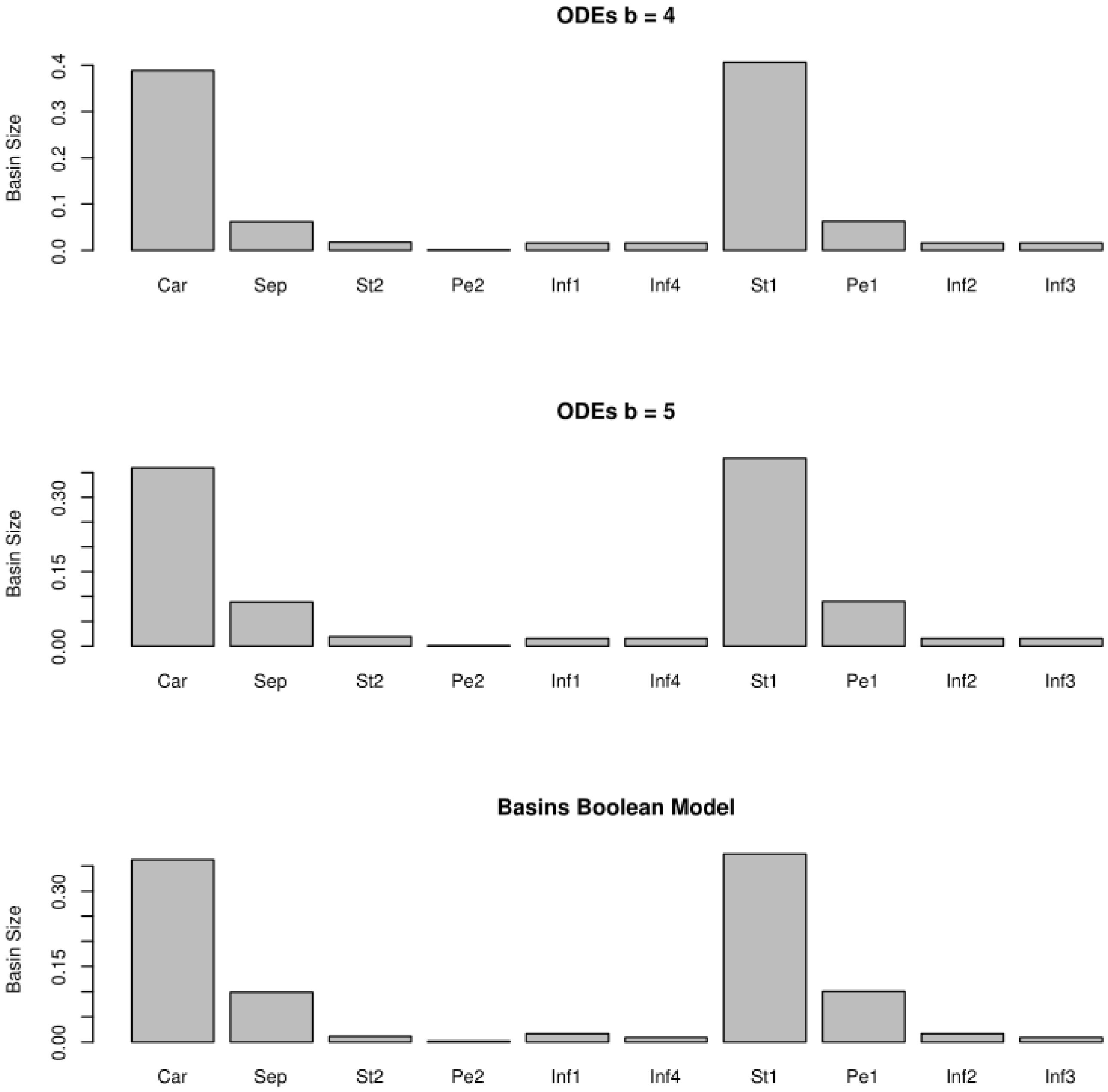

Figure A5. Comparison of the Attractors and Basins Uncovered with the Boolean and ODEs FOS-GRN Models.

Attractor_0	Perturbed_Gene	QualitativeChange	Attractor_Fin
Inf1	TFL1	1	SEP
Inf1	EMF1	1	Car
Inf2	TFL1	1	PE1
Inf2	UFO	1	Inf1
Inf2	EMF1	1	St1
Inf3	TFL1	1	St1
Inf3	UFO	1	Inf4
Inf3	EMF1	1	St1
Inf3	WUS	1	Inf2
Inf4	TFL1	1	Car
Inf4	EMF1	1	Car
Inf4	WUS	1	Inf1
Car	AG	1	Sep
Car	LFY	1	Other
Car	AP2	0	NA
Car	FT	0	NA
Car	PI	0	NA
Car	FUL	0	NA
Car	SEP	0	NA
Sep	AP1	1	Car
Sep	AP2	1	Other
Sep	LFY	0	NA
Sep	FT	0	NA
Sep	SEP	0	NA
St2	AG	1	Sep
St2	AP3	1	Car
St2	LFY	1	Other
St2	PI	1	Other
St2	SEP	1	Other
St2	AP2	0	NA
St2	FT	0	NA
St2	FUL	0	NA
Pe2	AP1	1	Car
Pe2	AP2	1	Other
Pe2	AP3	1	Sep
Pe2	LFY	1	Other
Pe2	PI	1	Sep
Pe2	SEP	1	Other
Pe2	FT	0	NA
St1	AG	1	PE1
St1	LFY	1	Other
St1	UFO	1	St2
St1	AP2	0	NA
St1	AP3	0	NA
St1	FT	0	NA
St1	FUL	0	NA
St1	PI	0	NA
St1	SEP	0	NA
St1	FUL	0	NA
Pe1	AP1	1	St1
Pe1	AP2	1	Other
Pe1	AP3	0	NA
Pe1	LFY	1	Other
Pe1	UFO	1	Pe2
Pe1	FT	0	NA
Pe1	PI	0	NA
Pe1	SEP	0	NA

Table A1. Summary of all the bifurcation analyses.

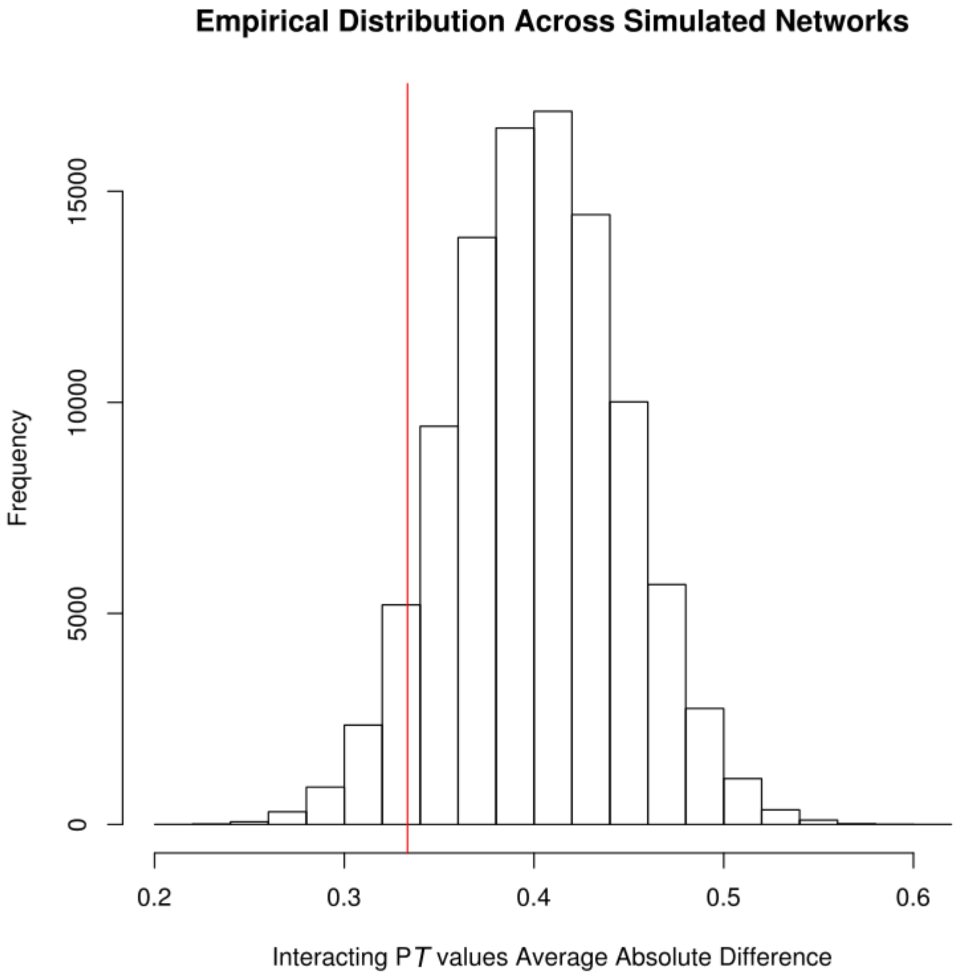

Figure A6. Histogram of the AAD in PT values calculated from simulated networks values.
