# Supplementary material for: Reshaping the epigenetic landscape during early flower development: induction of attractor transitions by relative differences in gene decay rates
Source: BMC Syst Biol. 2015 May 13;9:20. doi: 10.1186/s12918-015-0166-y (PMC4438470; doi:10.1186/s12918-015-0166-y)
Supplement: Additional file 2 — Results of the Bifurcation (Phase transition) Analysis. [file 12918_2015_166_MOESM2_ESM.pdf]

Additional File 2

Results of the Bifurcation (Phase transition) Analysis.

Set of graphs, one per each gene expressed in each attractor. In the graph we plotted the initial attractor state and its progressive change resulting from altering the decay parameter. This file included the graphs for all the 10 attractors (and their genes) in the following page order.

Carpel attractor (*Car*) - 2
Inflorescence 1 attractor (*Inf1*) - 3
Inflorescence 2 attractor (*Inf2*) - 4
Inflorescence 3 attractor (*Inf3*) - 5
Inflorescence 4 attractor (*Inf4*) - 6
Petal attractor 1 (*Pe1*) - 7
Petal attractor 1 (*Pe2*) - 8
Sepal attractor (*Sep*) - 9
Stamen attractor 1 (*St1*) - 10
Stamen attractor 2 (*St2*) - 11

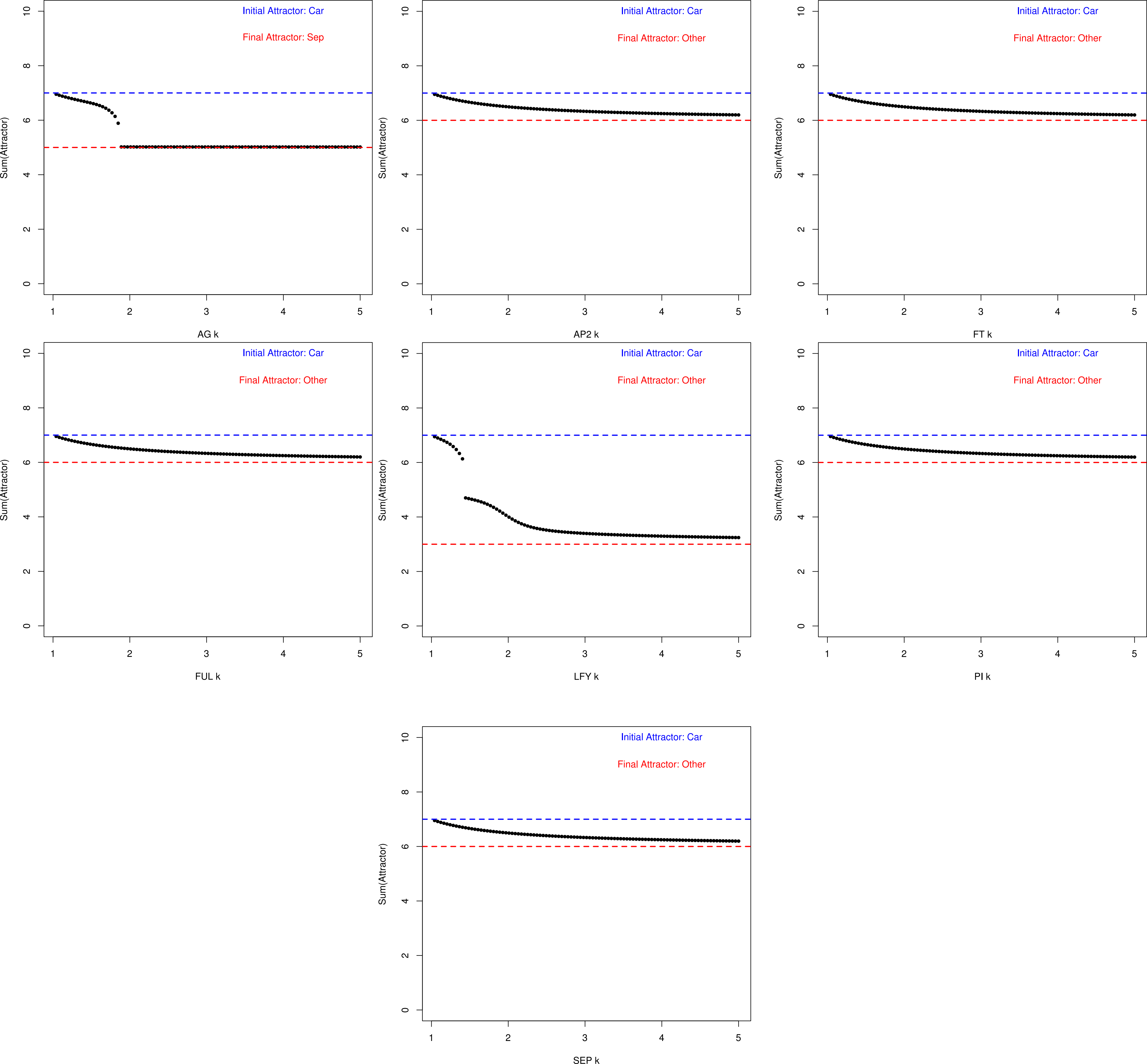
Initial Attractor: Car
Final Attractor: Sep
Sum(Attractor)
AG k
Initial Attractor: Car
Final Attractor: Other
Sum(Attractor)
AP2 k
Initial Attractor: Car
Final Attractor: Other
Sum(Attractor)
FT k
Initial Attractor: Car
Final Attractor: Other
Sum(Attractor)
FUL k
Initial Attractor: Car
Final Attractor: Other
Sum(Attractor)
LFY k
Initial Attractor: Car
Final Attractor: Other
Sum(Attractor)
PI k
Initial Attractor: Car
Final Attractor: Other
Sum(Attractor)
SEP k

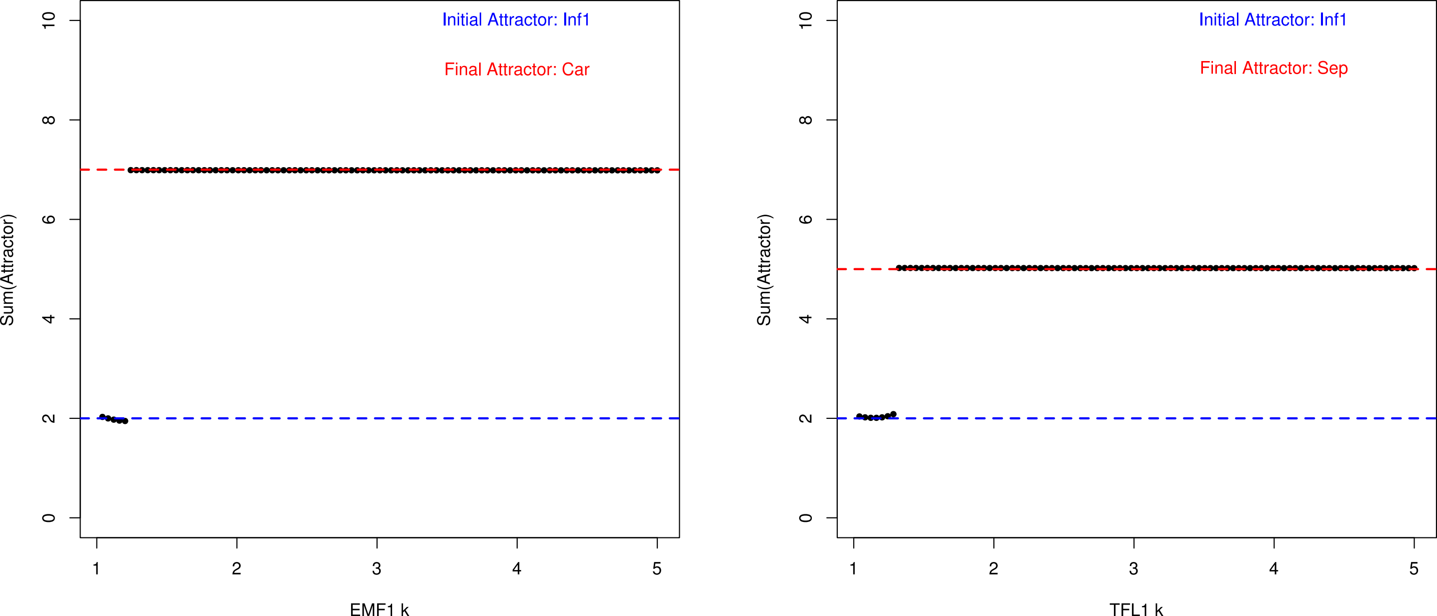
Initial Attractor: Inf1
Final Attractor: Car
Sum(Attractor)
EMF1 k
Initial Attractor: Inf1
Final Attractor: Sep
Sum(Attractor)
TFL1 k

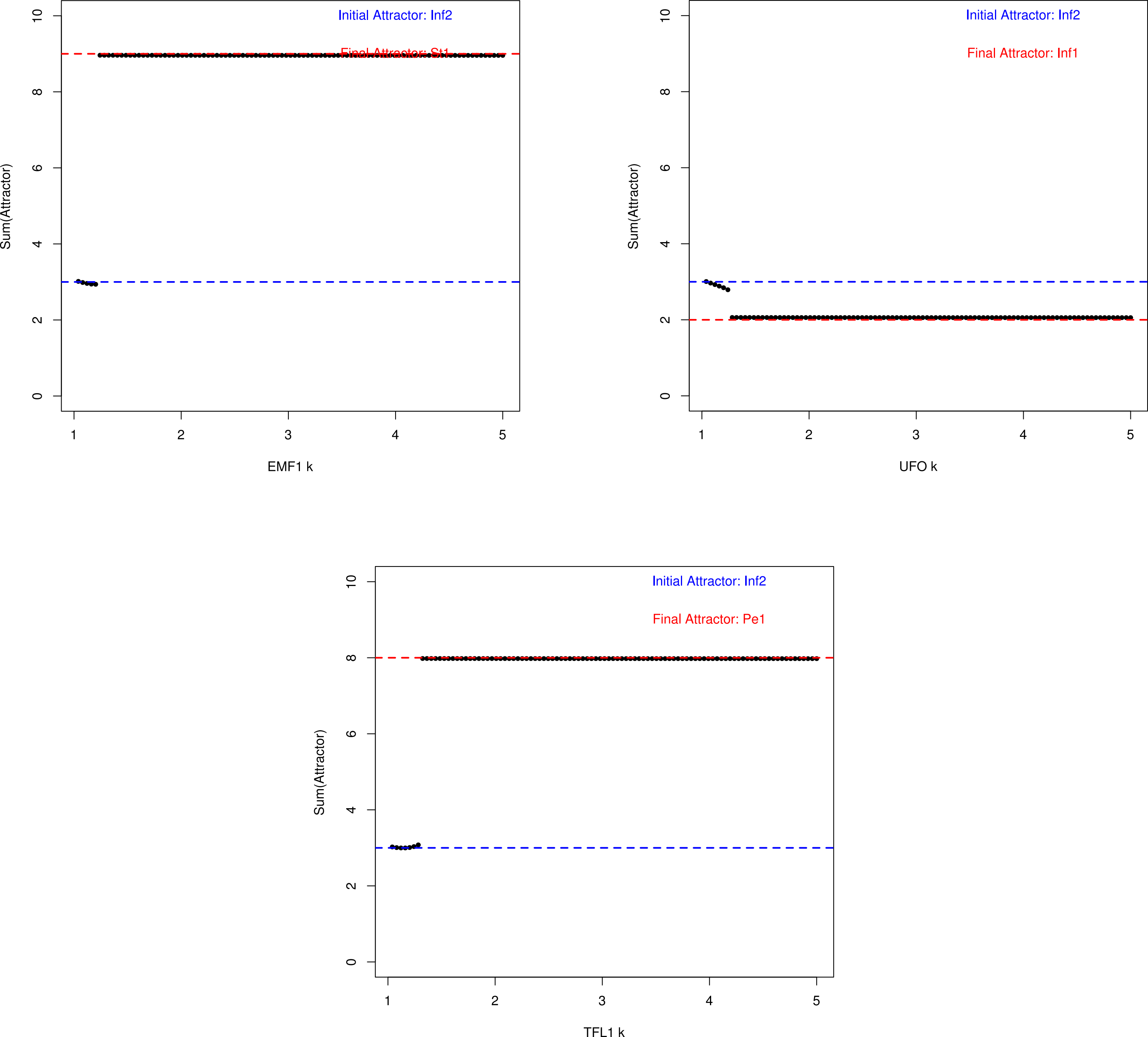
Initial Attractor: Inf2
Final Attractor: St1
Sum(Attractor)
EMF1 k
Initial Attractor: Inf2
Final Attractor: Inf1
Sum(Attractor)
UFO k
Initial Attractor: Inf2
Final Attractor: Pe1
Sum(Attractor)
TFL1 k

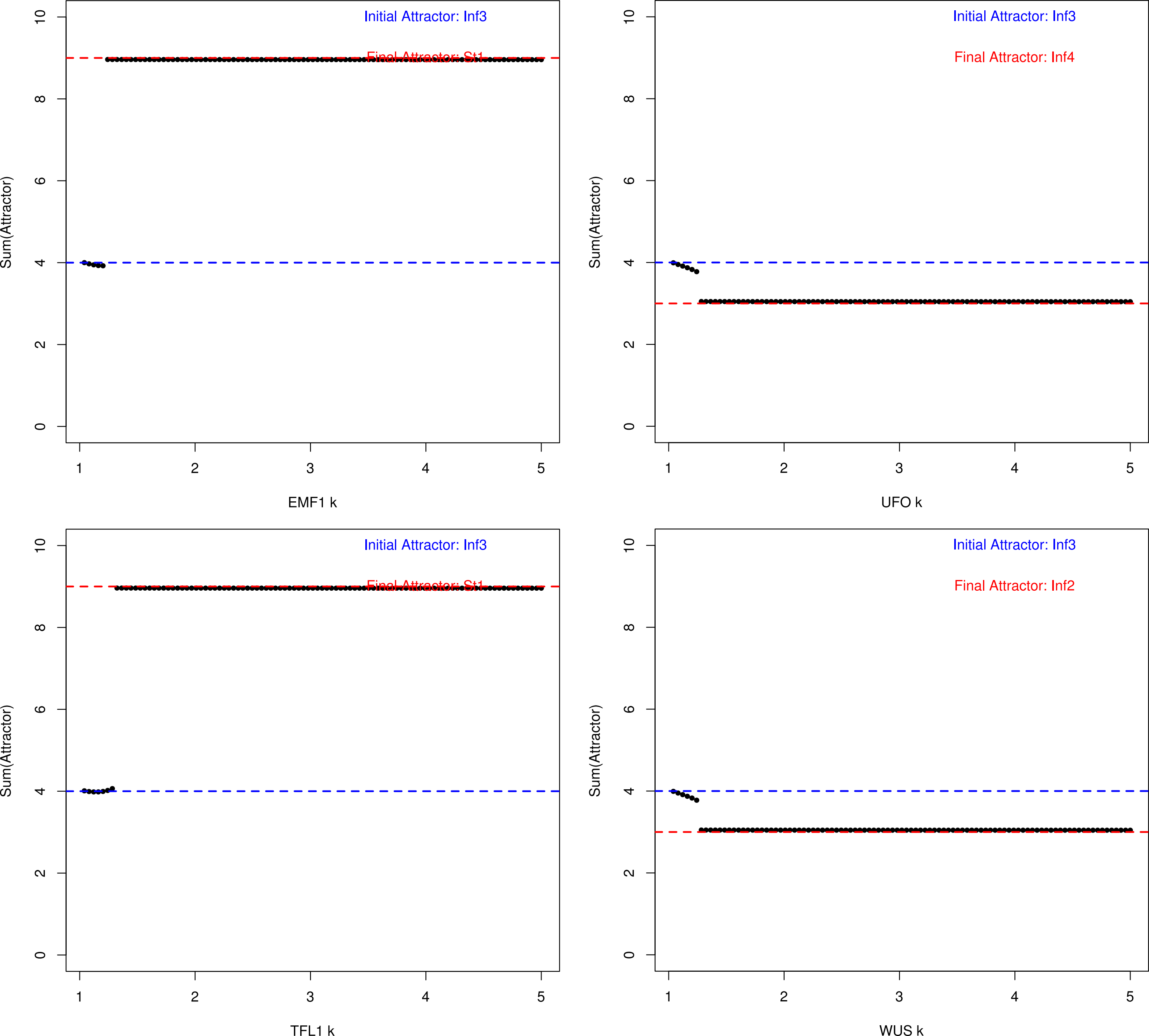
Initial Attractor: Inf3
Final Attractor: St1
Sum(Attractor)
EMF1 k
Initial Attractor: Inf3
Final Attractor: Inf4
Sum(Attractor)
UFO k
Initial Attractor: Inf3
Final Attractor: St1
Sum(Attractor)
TFL1 k
Initial Attractor: Inf3
Final Attractor: Inf2
Sum(Attractor)
WUS k
0
2
4
6
8
10
1
2
3
4
5

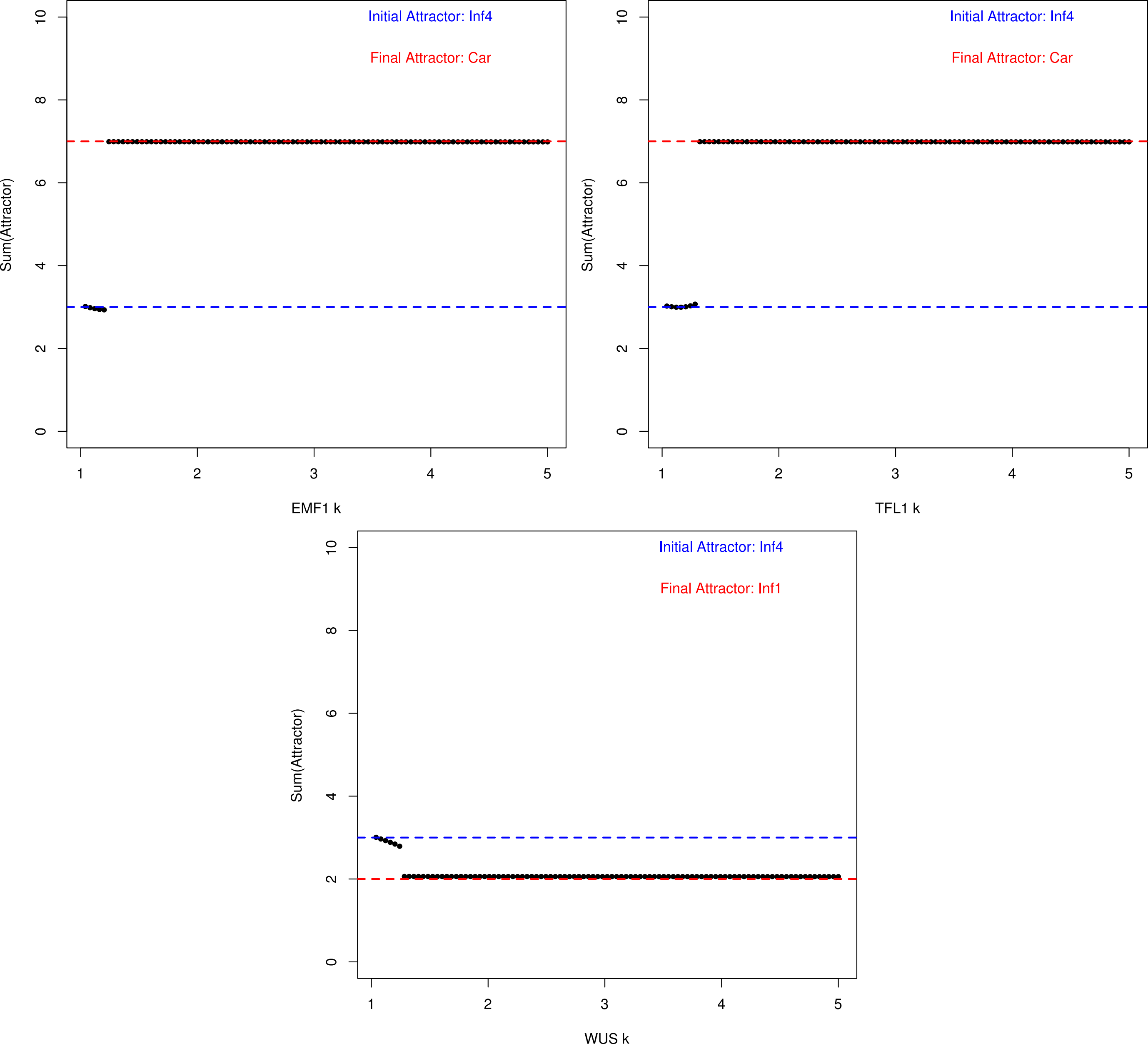

Initial Attractor: Inf4
Final Attractor: Car
Sum(Attractor)
0
2
4
6
8
10
1
2
3
4
5
EMF1 k
Initial Attractor: Inf4
Final Attractor: Car
Sum(Attractor)
TFL1 k
Initial Attractor: Inf4
Final Attractor: Inf1
Sum(Attractor)
WUS k

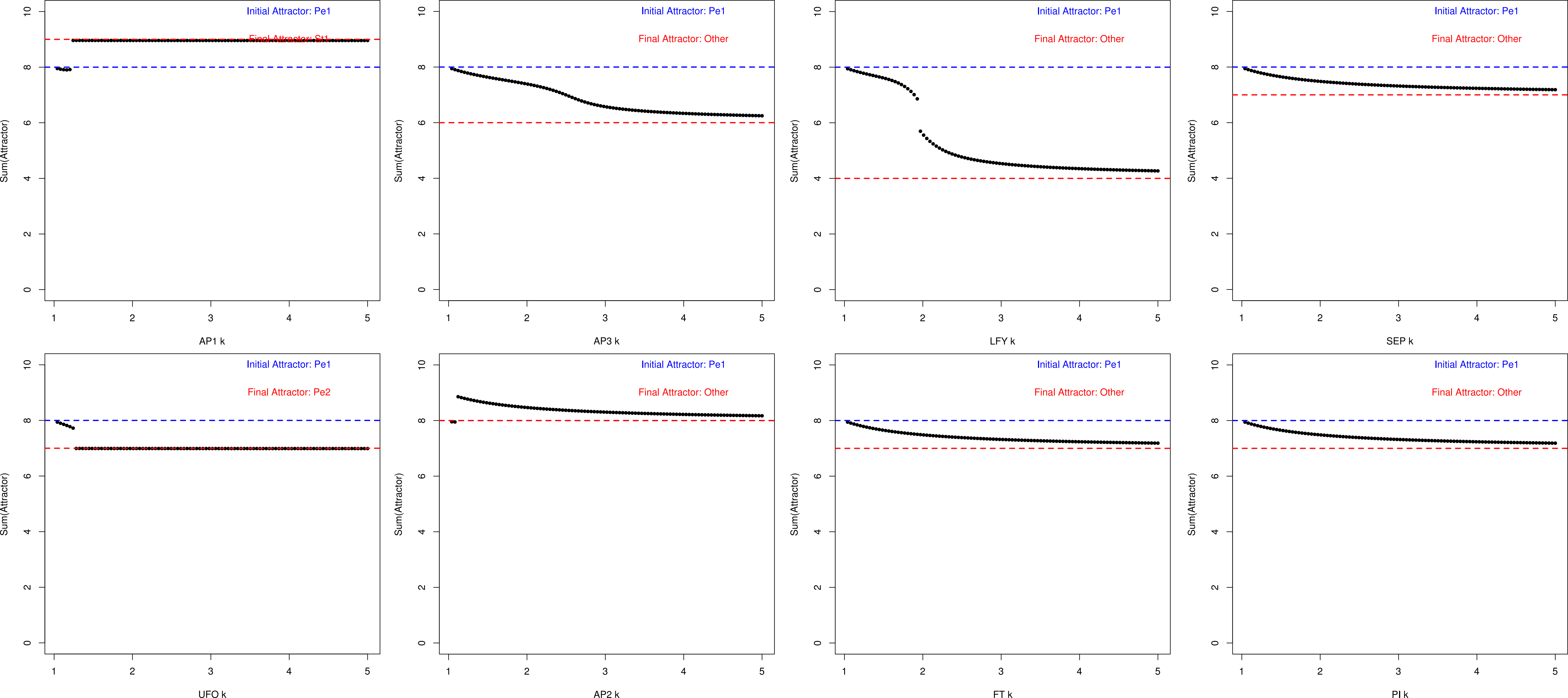
Initial Attractor: Pe1
Final Attractor: St1
Sum(Attractor)
AP1 k
Initial Attractor: Pe1
Final Attractor: Other
Sum(Attractor)
AP3 k
Initial Attractor: Pe1
Final Attractor: Other
Sum(Attractor)
LFY k
Initial Attractor: Pe1
Final Attractor: Other
Sum(Attractor)
SEP k
Initial Attractor: Pe1
Final Attractor: Pe2
Sum(Attractor)
UFO k
Initial Attractor: Pe1
Final Attractor: Other
Sum(Attractor)
AP2 k
Initial Attractor: Pe1
Final Attractor: Other
Sum(Attractor)
FT k
Initial Attractor: Pe1
Final Attractor: Other
Sum(Attractor)
PI k

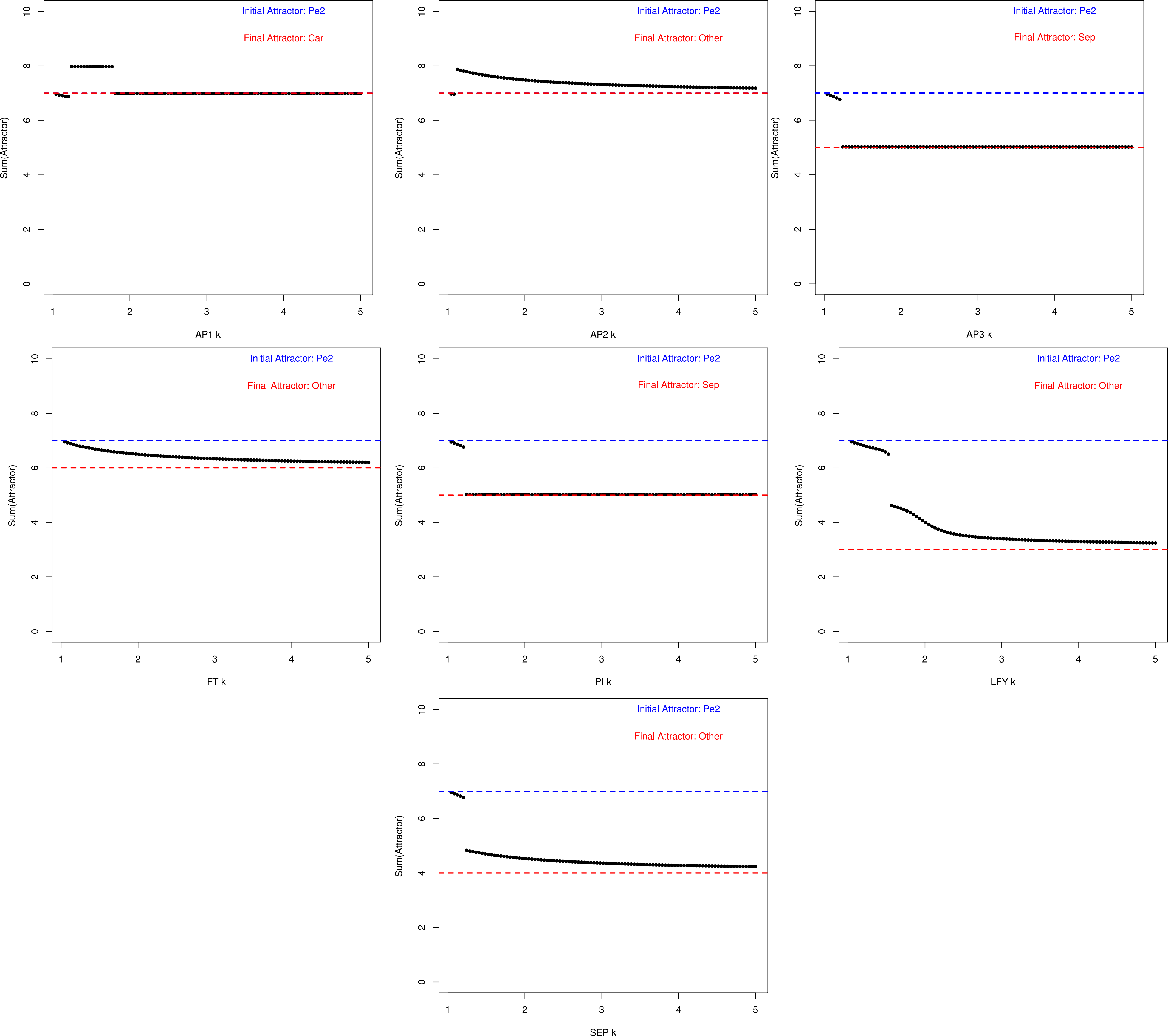

Initial Attractor: Pe2
Final Attractor: Car
Sum(Attractor)
AP1 k
Initial Attractor: Pe2
Final Attractor: Other
Sum(Attractor)
AP2 k
Initial Attractor: Pe2
Final Attractor: Sep
Sum(Attractor)
AP3 k
Initial Attractor: Pe2
Final Attractor: Other
Sum(Attractor)
FT k
Initial Attractor: Pe2
Final Attractor: Sep
Sum(Attractor)
PI k
Initial Attractor: Pe2
Final Attractor: Other
Sum(Attractor)
LFY k
Initial Attractor: Pe2
Final Attractor: Other
Sum(Attractor)
SEP k

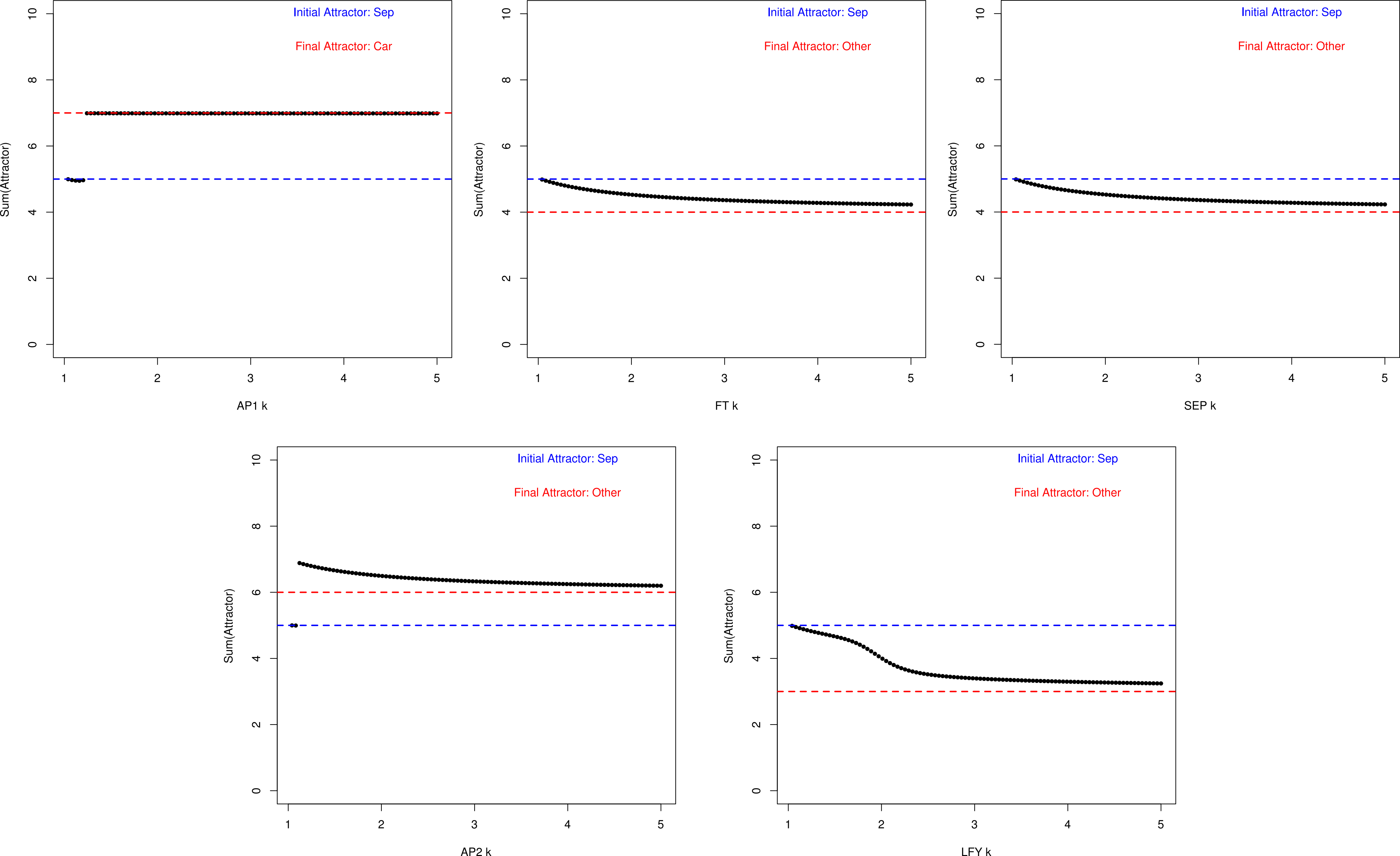
Initial Attractor: Sep
Final Attractor: Car
Sum(Attractor)
AP1 k
Initial Attractor: Sep
Final Attractor: Other
Sum(Attractor)
FT k
Initial Attractor: Sep
Final Attractor: Other
Sum(Attractor)
SEP k
Initial Attractor: Sep
Final Attractor: Other
Sum(Attractor)
AP2 k
Initial Attractor: Sep
Final Attractor: Other
Sum(Attractor)
LFY k

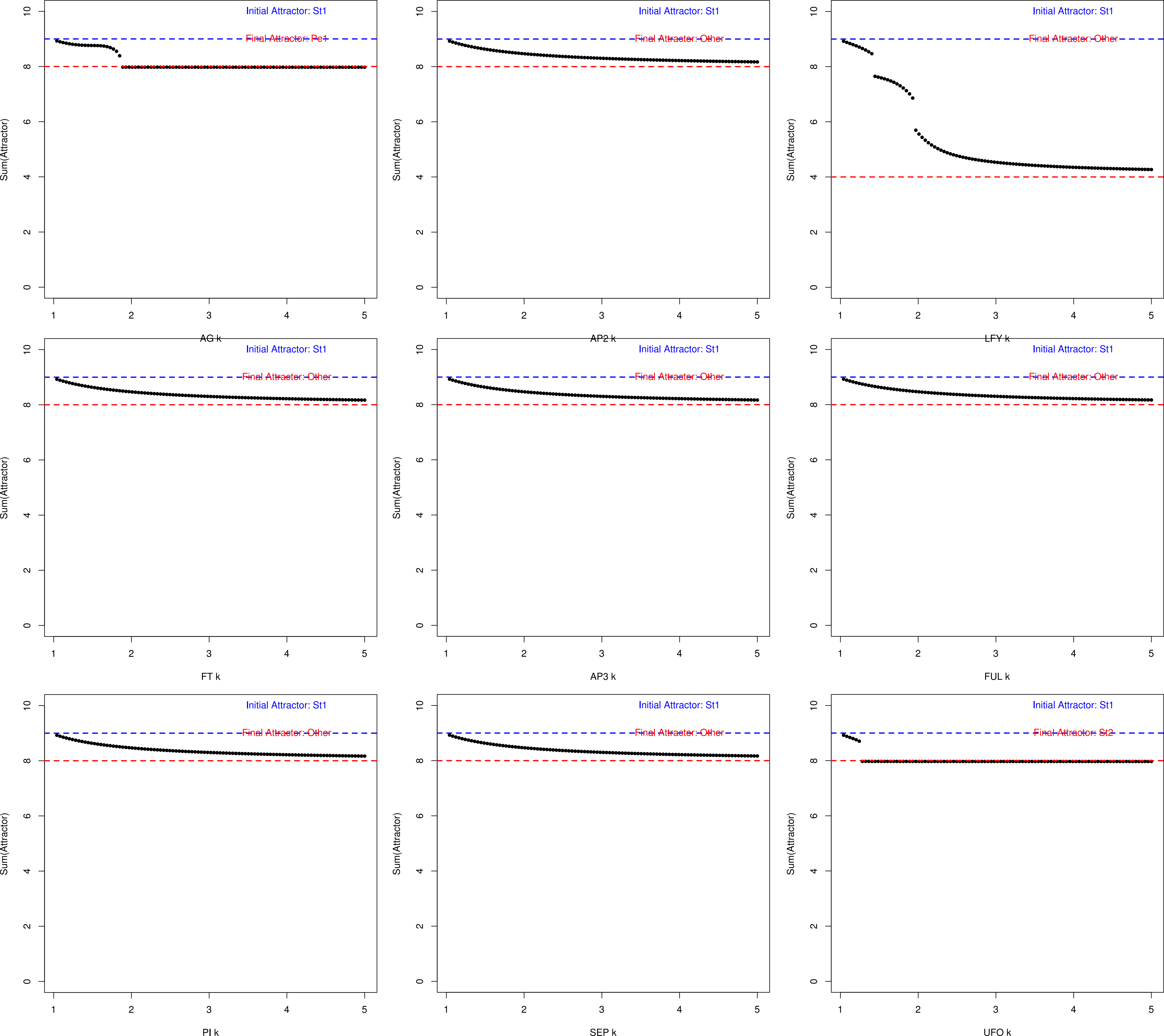

Initial Attractor: St1
Final Attractor: Pe1
Sum(Attractor)
AG k
Initial Attractor: St1
Final Attractor: Other
Sum(Attractor)
AP2 k
Initial Attractor: St1
Final Attractor: Other
Sum(Attractor)
LFY k
Initial Attractor: St1
Final Attractor: Other
Sum(Attractor)
FT k
Initial Attractor: St1
Final Attractor: Other
Sum(Attractor)
AP3 k
Initial Attractor: St1
Final Attractor: Other
Sum(Attractor)
FUL k
Initial Attractor: St1
Final Attractor: Other
Sum(Attractor)
PI k
Initial Attractor: St1
Final Attractor: Other
Sum(Attractor)
SEP k
Initial Attractor: St1
Final Attractor: St2
Sum(Attractor)
UFO k

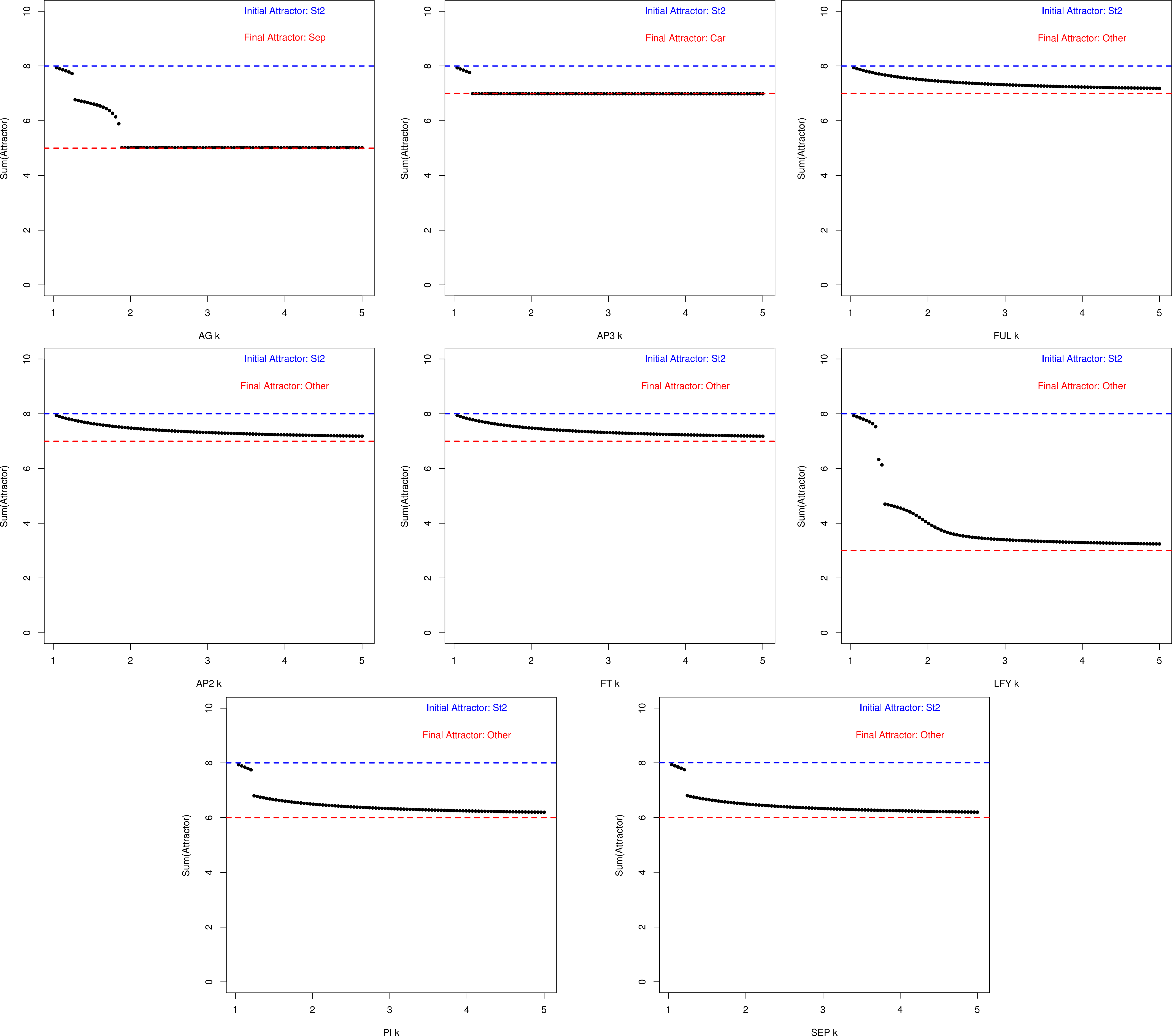

Initial Attractor: St2
Final Attractor: Sep
Sum(Attractor)
AG k
Initial Attractor: St2
Final Attractor: Car
Sum(Attractor)
AP3 k
Initial Attractor: St2
Final Attractor: Other
Sum(Attractor)
FUL k
Initial Attractor: St2
Final Attractor: Other
Sum(Attractor)
AP2 k
Initial Attractor: St2
Final Attractor: Other
Sum(Attractor)
FT k
Initial Attractor: St2
Final Attractor: Other
Sum(Attractor)
LFY k
Initial Attractor: St2
Final Attractor: Other
Sum(Attractor)
PI k
Initial Attractor: St2
Final Attractor: Other
Sum(Attractor)
SEP k
